# Transnasal Administration of Surfactant Aerosol to the Lungs of Infants Using Direct Interfaces – Lessons Learned from Computational Fluid Dynamics

**DOI:** 10.1007/s11095-026-04186-1

**Published:** 2026-09-03

**Authors:** Hasan Jubaer, Dale Farkas, Caleb Dalton, Mohammad A. M. Momin, Michael Hindle, Worth Longest

**Affiliations:** 1 Department of Mechanical and Nuclear Engineering, Virginia Commonwealth University, 401 West Main Street, P.O. Box 843015, Richmond, VA 23284-3015 USA; 2 Department of Pharmaceutics, Virginia Commonwealth University, Richmond, VA, USA

**Keywords:** aerosol surfactant therapy, air-jet DPI, CFD, nose-to-lung aerosol delivery, particle deposition, preterm infant, respiratory drug delivery

## Abstract

**Purpose:**

Direct-to-infant (D2I) transnasal aerosol surfactant therapy (AST) offers a promising non-invasive alternative for treating neonatal respiratory distress syndrome (RDS). However, achieving high lung delivery efficiency with dry powder inhalers (DPIs) requires careful optimization of complex fluid–particle interactions. This study used computational fluid dynamics (CFD) to optimize an Infant Air‑Jet Dry Powder Aerosol Delivery System (iDP‑ADS) and derive transferable design principles.

**Methods:**

A CFD model of the iDP‑ADS coupled to a 31‑week preterm nose‑throat geometry was validated against *in vitro* measurements. Sensitivity analyses evaluated modeling assumptions (steady‑state vs. transient, monodisperse vs. polydisperse) and critical design parameters influencing aerosol delivery, such as upstream air volume, interface geometry, temporal aerosol emission profiles, and actuation flow rates.

**Results:**

The CFD model demonstrated strong *in vitro* agreement (2.8% mean absolute difference). Regarding modeling: standard simplifications, particularly steady-state approximations, overpredicted lung transmission (~24%), establishing the necessity of high-fidelity transient simulations with polydisperse aerosols. Regarding aerosol delivery: integrating an upstream air volume created "hydrodynamic shielding," reducing relative interface losses by up to 50%. A 10 mm diffusive path length and 20° (inward and downward) prong alignment balanced jet dissipation while minimizing nasal deposition. Front-loading of aerosol emission prevented entrapment of 10–14% of the dose in anatomical dead space. An optimal system-specific actuation flow rate of 3 L/min achieved ~53.5% aerosol lung transmission, balancing aerosolization efficiency against turbulence-driven transport losses.

**Conclusions:**

High-efficiency transnasal AST requires balancing often competing aerosol generation physics, transport behavior, and clinical constraints. This study highlights CFD as a vital design tool, providing key lessons to inform future modeling and practical interface development.

**Graphical Abstract:**

**Figure:**
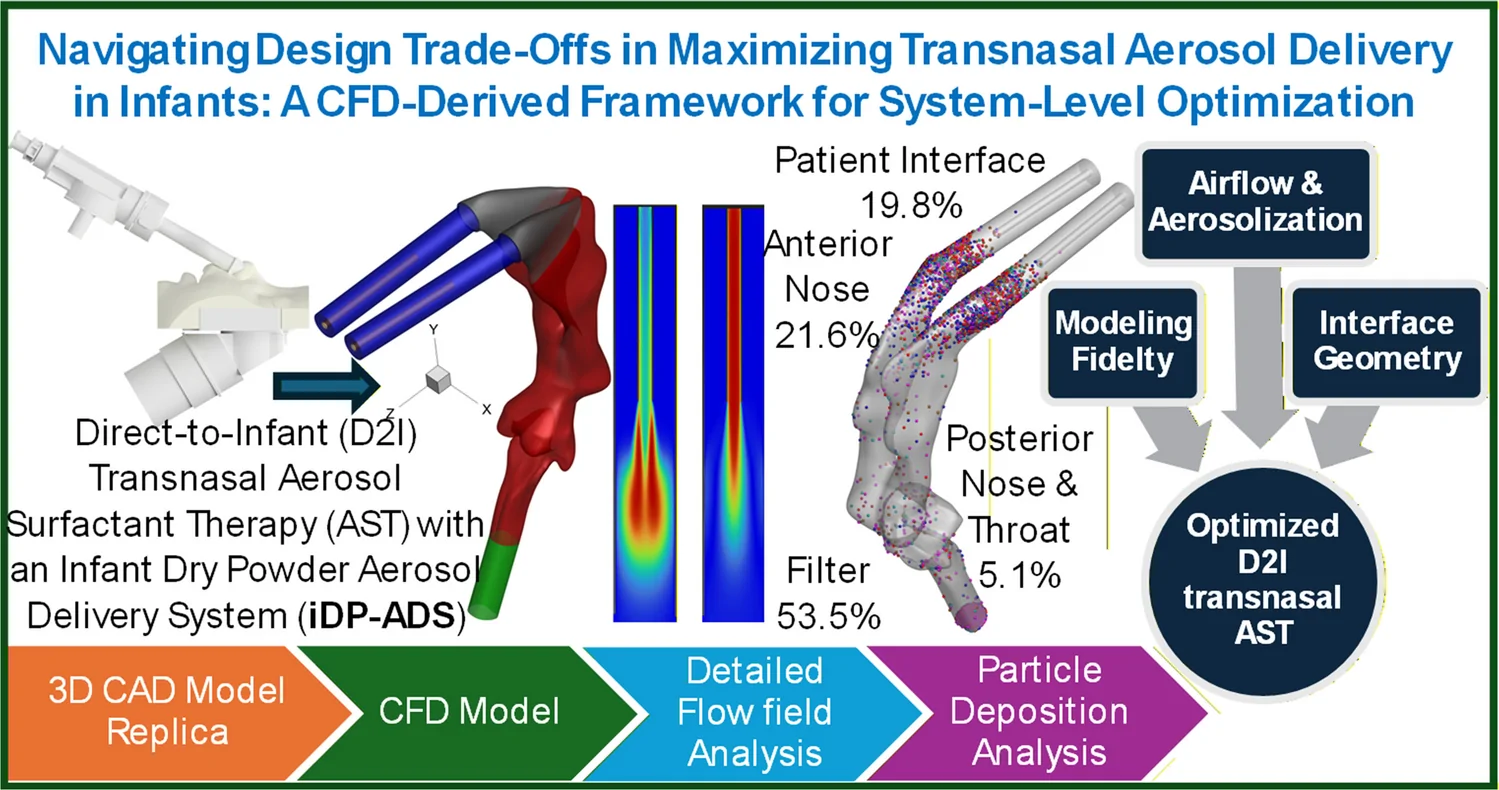

**Supplementary Information:**

The online version contains supplementary material available at https://doi.org/10.1007/s11095-026-04186-1.

## Introduction

Transnasal aerosol administration targeting the lower airways has gained broad acceptance in acute care settings, based on its non-invasive nature, patient comfort, and suitability across all ages [1]. By exploiting the lung’s extensive surface area and the thin epithelial lining [2], this strategy delivers superior therapeutic concentrations within the lungs compared to oral or intravenous routes, while limiting off-target exposure and systemic side effects [3]. The rationale for transnasal aerosol administration is particularly compelling in infants, who are predominantly nasal breathers [4–8]. Furthermore, diverse therapeutics ranging from small molecules to complex biologics can be delivered transnasally [1] to manage prevalent respiratory conditions—including respiratory distress syndrome (RDS), acute lung injury, pneumonias, and bronchiolitis [9–13]—that account for substantial morbidity in neonatal care. RDS alone, caused by surfactant insufficiency, is responsible for approximately 15% of neonatal deaths globally [14, 15].

While surfactant replacement therapy remains the established clinical standard for RDS [16], all currently approved techniques require intubation to instill relatively large liquid volumes (1.2–4 mL/kg) directly into the lungs [9, 16–18]. Besides procedural risks associated with delivery tube placement, liquid bolus instillation is frequently accompanied by complications such as airway obstruction from surfactant reflux, alveolar flooding, oxygen desaturation, bradycardia, hemodynamic instability, and reductions in cerebral blood flow [19–24]. Moreover, liquid boluses often distribute unevenly, causing ventilation-perfusion mismatch, reduced compliance and potential alveolar damage and/or inflammation [25–29].

Aerosol Surfactant Therapy (AST) aims to avoid these complications by enabling administration during spontaneous breathing or non-invasive ventilation [9, 17, 19], potentially permitting earlier intervention, lower dose and associated cost reductions, and expanded access in low-resource settings [26, 30]. However, realizing the potential of AST requires overcoming delivery challenges inherent to neonatal airways and delivery platforms [7, 8, 31]. Among available platforms, dry powder inhalers (DPIs) offer potential advantages, including formulation stability, low device cost, rapid high-dose administration, and high-efficiency lung delivery via small particle generation and controlled excipient enhanced growth (EEG) technology [32–39]. Recent *in vivo* studies in neonatal-sized (≈1600 g) rabbit models support this approach. DiBlasi, *et al*. [25] demonstrated that a dry powder synthetic lung surfactant (SLS) administered through an endotracheal tube elicited a physiological response equivalent to liquid surfactants but at an order of magnitude lower dose, with less lung injury and more uniform aeration. Similarly, Longest, *et al*. [40] reported improved efficacy at lower doses and an order of magnitude faster response time than liquid instillation.

To translate this potential into a non-invasive RDS treatment, our group is developing an infant Dry Powder Aerosol Delivery System (iDP-ADS) for transnasal AST administration. Our previous study coupling the iDP-ADS with nasal Continuous Positive Airway Pressure (nCPAP) systems demonstrated 37–60% lung transmission with low inter-subject variability [41]. Although clinically valuable, dependence on specific CPAP systems may limit global applicability. Because aerosol delivery could be completed in under one minute [40], a direct-to-infant (D2I) transnasal AST approach was proposed [42], in which nCPAP is briefly (approximately 4–8 breaths) interrupted during administration, while the iDP-ADS maintains positive pressure (a PIP of ≤ 25 ± 2 cm H_2_O and a PEEP of 6 ± 2 cm H_2_O) via a Pressure Monitoring and Control (PMC) unit and minimizes humidity exposure of the SLS-EEG powder. Lung delivery efficiencies of ~ 55% were achieved with a model EEG formulation.

A series of studies refined the techniques and components necessary for the D2I transnasal platform. Bass, *et al*. [43] utilized Computational Fluid Dynamics (CFD) to identify the flow turbulence exiting the nasal prongs as a primary driver of aerosol loss in the nose-throat (NT) region. Howe, *et al*. [44, 45] evaluated several patient interfaces (PI), achieving lung delivery efficiencies of 50–60%, robust to variations in loaded dose and downstream pulmonary mechanics, using both a spray-dried SLS-EEG formulation and a surrogate.

Despite aerosol lung transmissions approaching 60% of device loaded dose, previous studies relied on either model EEG formulations or SLS-EEG powder produced using low-output spray dryers. Production scale-up modestly increased particle size that surprisingly caused substantial declines in lung transmissions. Current testing indicates interface losses exceeding 20% and NT losses reaching 40%, reducing the predicted lung transmission to ~ 25%. The higher targeted lung pressures of the D2I approach relative to the nCPAP-coupled delivery further reduced lung delivery efficiency. Although previous bifurcation and co-flow designs [42] partially mitigated these losses, the underlying transport physics could not be resolved experimentally, motivating a more in-depth investigation and understanding.

With the SLS-EEG formulation and aerosol size largely fixed, performance improvement must come from controllable parameters related to the device configuration and flow dynamics, e.g., the actuation flow rate (2–6 L/min), the temporal emission profile, and the PI design and orientation. To guide the optimization efforts, a target product profile (TPP) has been established, benchmarked against a reference 1500 g VCU/DiBlasi preterm infant model [43, 46]. Key elements of this TPP include a target for extrathoracic loss of ≤ 40% (minimal) or ≤ 30% (optimal), and a lung delivery efficiency of ≥ 25% (minimal) or ≥ 50% (optimal), based on the device-loaded dose. Because the interface contributes substantially to both deposition and aerosol growth, interface optimization is a critical design objective.

Meeting these TPP targets requires a fundamental understanding of the fluid-particle dynamics that *in vitro *experiments alone cannot provide. CFD offers a robust platform to visualize turbulence and secondary flow structures that drive depositional losses [43, 47], and captures complex particle transport during therapeutic aerosol delivery to guide optimization strategies [48–52]. A "digital twin" of the system lets variables be systematically isolated and optimized, but its predictive utility is intrinsically linked to its fidelity. To manage computational costs, respiratory simulations routinely employ simplifying assumptions such as idealized fixed geometries, steady‑flow conditions, and monodisperse particle distributions [53, 54]. These simplifications may not accurately capture the transient, high-velocity physics of the iDP-ADS.

Thus, the remaining barriers to achieving the TPP involve: (i) establishing whether common modeling simplifications provide sufficient predictive accuracy; and (ii) identifying and evaluating controllable device and operating parameters that influence flow-field development, aerosol transport, and depositional losses.

The objective of this study is to first develop an *in vitro*-validated CFD model of D2I transnasal AST using an SLS-EEG aerosol delivered with the iDP-ADS to a neonate, and then implement this in silico model to assess the impact of controllable parameters with the goal of satisfying the TPP. Beyond optimizing lung delivery performance, the distinct focus of this study will be to synthesize the key lessons learned during CFD-guided design and refinement of the iDP-ADS regarding: (i) the impact of modeling simplifications, including steady-state simulations, steady particle injection, and monodisperse particle distributions (CFD modeling lessons); and (ii) how controllable design parameters—such as interface geometric optimization (via variation in length, ergonomic alignment, or the introduction of an upstream volume), actuation flow rate adjustments, and temporal aerosol emission profiles—can be leveraged to move performance toward the TPP (aerosol delivery lessons).

## Materials and Methods

### Experimental Methods

#### Formulation Materials

The SLS-EEG powder formulation used in this study consisted of 57.5% dipalmitoylphosphatidylcholine (DPPC), 7.5% 1-palmitoyl-2-oleoyl-sn-glycero-3-phosphoglycerol (POPG), 9% mannitol, 6% sodium chloride, and 20% L-leucine (w/w). The formulation was prepared via spray drying using a Büchi S-300 Mini Spray Dryer (Büchi Labortechnik AG, Switzerland) according to methods previously described by Strickler, *et al*. [55]. Regarding the source of the ingredients of the formulation, DPPC and POPG were purchased from Avanti Polar Lipids, Inc. (Alabaster, AL, USA); L-leucine was obtained from Sigma-Aldrich Chemical Co. (St. Louis, MO, USA); and sodium chloride and mannitol were acquired from Fisher Scientific Co. (Hanover Park, IL, USA).

## Device Design and Assembly

The iDP-ADS consists of a positive-pressure air source, an inlet airflow passage, an aerosolization chamber, an outlet capillary, and a patient interface (PI) comprising a diffusional pathway and nasal prongs [33]. High-velocity air boluses actuated into the chamber drive the aerosolization of the powder via air-jet expansion and secondary velocity currents [56, 57]. The outlet capillary ensures that only sufficiently deaggregated particles enter the diffusional flow passage. This passage reduces velocity and turbulence before the nasal prongs, whose geometry significantly influences nasal depositional losses and lung transmission [43, 44].

**Fig. 1:**
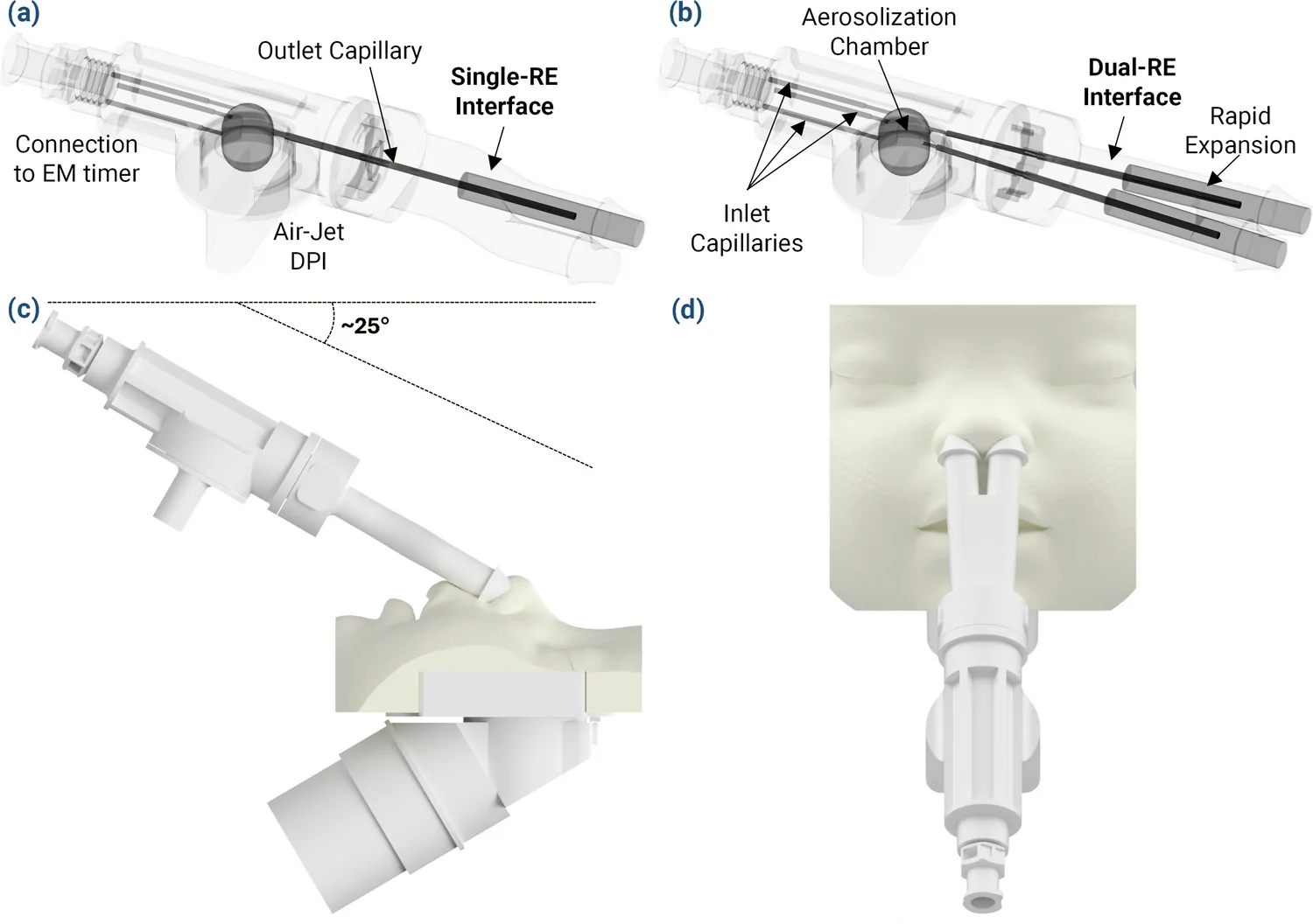
Overview of the direct-to-infant transnasal aerosol delivery setup attached to a preterm infant nasal model. Panels (**a**) & (**b**) show translucent isometric views of the 3D rendered infant air-jet DPI with patient interfaces used for the aerosol delivery: (**a**) Air-jet DPI device attached to a single-RE interface, (**b**) Air-jet DPI device attached to a dual-RE interface. Panels (**c**) and (**d**) illustrate the side and top views of the setup, respectively.

The iDP-ADS units used in this study were 3D printed using Formlabs 3/3B + SLA printers (Formlabs Inc., Somerville, MA, USA) and Formlabs Clear V4 SLA resin. Metal (stainless steel) capillary tubes of appropriate sizes were used for aerosol outlets of the aerosolization chamber. For initial aerosol characterization and emission profile analysis, the devices were assembled without a PI. For a complete assembly of the iDP-ADS (Fig. 1a and b), two different PIs were used. Each design incorporated a rapid expansion (RE) type diffusional pathway to transition from the outlet capillary to the inlet of the anterior nose. The single-RE interface utilized a 0.89 mm internal diameter (ID) outlet capillary. In contrast, the dual-RE interface consisted of two 0.6 mm ID capillaries that subsequently increased to 0.89 mm ID upstream of the aerosol exit. Nasal prongs of the RE interfaces were positioned in the nostrils as shown in Fig. 1c and d.

## Characterization of the Aerosol Emission Profile

**Fig. 2:**
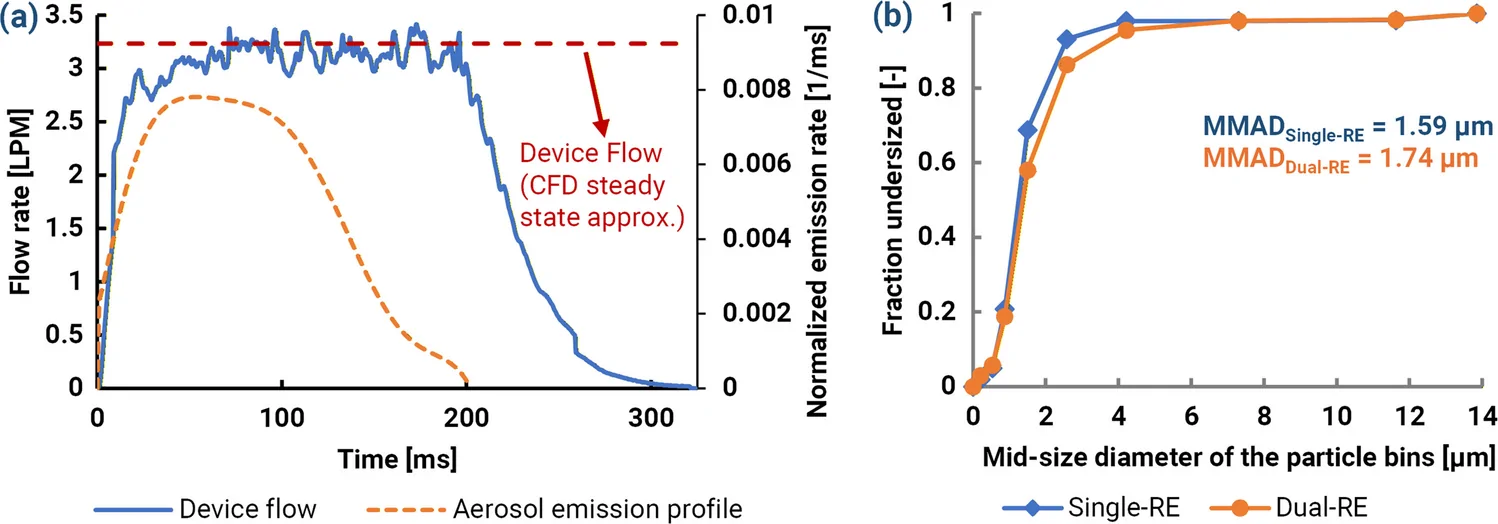
(**a**) Recorded flow rates and aerosol emission rates during the *in vitro* trials of the transnasal aerosol delivery systems operated at a target peak flow rate of 3 L/min, alongside the steady state approximation of the device flow rate used for CFD simulations. (**b**) Cumulative distribution of the initial particle sizes measured experimentally and used in the CFD simulations of the aerosol delivery to the infant nose-throat model.

The aerosol produced by the air-jet DPI device was characterized by laser diffraction using the Spraytec system (Malvern Panalytical, UK). The DPI device was mounted on a ring stand, positioned 6 cm from the detector and 1.5 cm from the beam edge. While testing the dual outlet device, in which aerosol is emitted from both capillary outlets, the aerosol from one of the outlets was directed away from the measurement zone and emission from only a single outlet capillary was considered. The device orientation was adjusted so that the capillary was horizontal and aligned with the center of the laser beam. The aerosolization chamber of the iDP-ADS was loaded with 5 mg of powder inside a humidity-controlled chamber (relative humidity < 20%). Actuation was performed using an electronic actuator (electromechanical; EM Timer) delivering air at a target peak flow rate of 3 L/min. The total actuated air volume was approximately 11.5 mL, monitored by a Sensirion SFM3400 Airflow Sensor (Sensirion AG, Stäfa, Switzerland). The corresponding device flow rate profile is shown in Fig. 2a (blue line).

Laser diffraction measurements were conducted in an open-bench configuration without vacuum assistance. Using a 300 mm lens and a laser-to-detector distance of approximately 23 cm, data were acquired at 1 kHz during 1.0 s events, triggered when transmission dropped below 99%. Acquisition included 10 ms of pre-trigger data. Automatic alignment and 10 s background measurements were performed before each actuation to reset light, dark, and electronic signals. All detector rings were used for data capture; however, rings 1–15 were excluded during post-processing to minimize beam steering artifacts.

For estimating aerosol mass emission profiles, only the transmission (calculated as 100% minus the obscuration percentage) data from the detailed measurements of the particle size distributions were utilized. It was assumed that the aerosol mass emission rate was inversely proportional to measured transmission. The area under the obscuration curve was normalized to represent 100% of the aerosolized drug mass and discretized into small time steps to calculate the emission rate throughout actuation. This profile (see Fig. 2a) was then fitted with polynomial equations, which were subsequently used to generate injection files for CFD simulations. This ensured that the simulations accurately reflected the time-dependent variation in particle number and mass during actuation.

## *In Vitro *Experiments

To characterize the aerosol output for sizing and subsequent deposition studies, the aerosolization chamber of the iDP-ADS was loaded with 5 mg of SLS-EEG powder and actuated with a square waveform flow profile at a rate of 3 L/min, controlled via an EM timer. The aerosol particle size distribution (PSD) exiting the capillaries of both configurations (single-RE and dual-RE) was determined using a Next Generation Impactor (NGI) (MSP, TSI, Shoreview, MN, USA) following methods described previously [35]. The device outlet was positioned at the open inlet of the NGI and makeup air was used to entrain the aerosol into the 45 L/min flow entering the preseparator of the NGI. The cutoff diameters of each NGI stage at the operating flow rate were determined in accordance with USP < 601 > Apparatus 5 [58]. Impactor stages were coated with MOLYKOTE® 316 silicone spray (Dow Corning, Midland, MI, USA) to minimize particle bounce. Following aerosolization, formulation mass retained in the device as well as formulation deposited on the pre-separator, impaction plates, and NGI filter were recovered and quantified by high-performance liquid chromatography (HPLC) analysis [40, 59].

To calculate the aerosol characteristics, as described by Farkas, *et al*. [60], the cumulative percentage of the drug mass was plotted against the corresponding stage cut-off diameters. Linear interpolation of this curve was then used to derive the mass median aerodynamic diameter (MMAD), the fine particle fraction ($FP{F}_{<5 \mu m}$), and the sub-micron fraction ($FP{F}_{<1 \mu m}$). These FPF metrics are defined as the mass of particles with aerodynamic diameters below 5 μm and 1 μm, respectively, normalized as a percentage of the total emitted dose. Furthermore, the polydispersity of each aerosol size distribution was quantified using the span, which is defined as follows:

1 $$span=\frac{{d}_{90}-{d}_{10}}{{d}_{50}}$$

where ${d}_{90}, {d}_{50}, \text{and } {d}_{10}$ are the diameters below which 90%, 50%, and 10% of the particle mass fall, respectively. These three characteristic diameters were determined by the same method of linear interpolation as the MMAD, and thus ${d}_{50}$ is identical to the MMAD.

The experimental setup for deposition studies of D2I transnasal AST, including the iDP-ADS and the preterm NT model, is illustrated in Fig. 1c and d. A realistic NT model of a 31-week gestational age (1500 g) preterm infant (designated as the “Intermediate” model in the preterm infant model series introduced by Jubaer, *et al*. [41]) was used to determine *in vitro* NT deposition of the aerosol. This NT model is split into two sections to separate the anterior region from the middle passage and nasopharynx. During SLS-EEG aerosol administration, the nasal interface was inserted into the nostrils of the NT model at an approximate 25° angle to the horizontal (Fig. 1c) and held steady while the device was actuated. A Pulmoguard II™ filter (Queset Medical, North Easton, MA, USA) was attached to the tracheal outlet of the NT model to capture the aerosol fraction representing the lung transmission. Following administration, the components of the experimental setup were disassembled, and deposited formulation mass in each component was recovered for quantitative analysis.

All deposition samples were recovered by dissolution in an appropriate volume of methanol and assayed to determine the mass of DPPC using a validated HPLC method [40, 59]. Samples were analyzed on a Waters Alliance 2695 Separation Module with a photodiode array UV detector (Waters Corporation, Milford, MA, USA). A 50 μL injection volume was used, and separation was achieved on a Waters XSelect CSH C18 XP column (2.5 μm, 4.6 × 100 mm) operated at a flow rate of 0.8 mL/min and maintained at 45°C.

### CFD Simulations

#### Computational Domains and Boundary Conditions

**Fig. 3:**
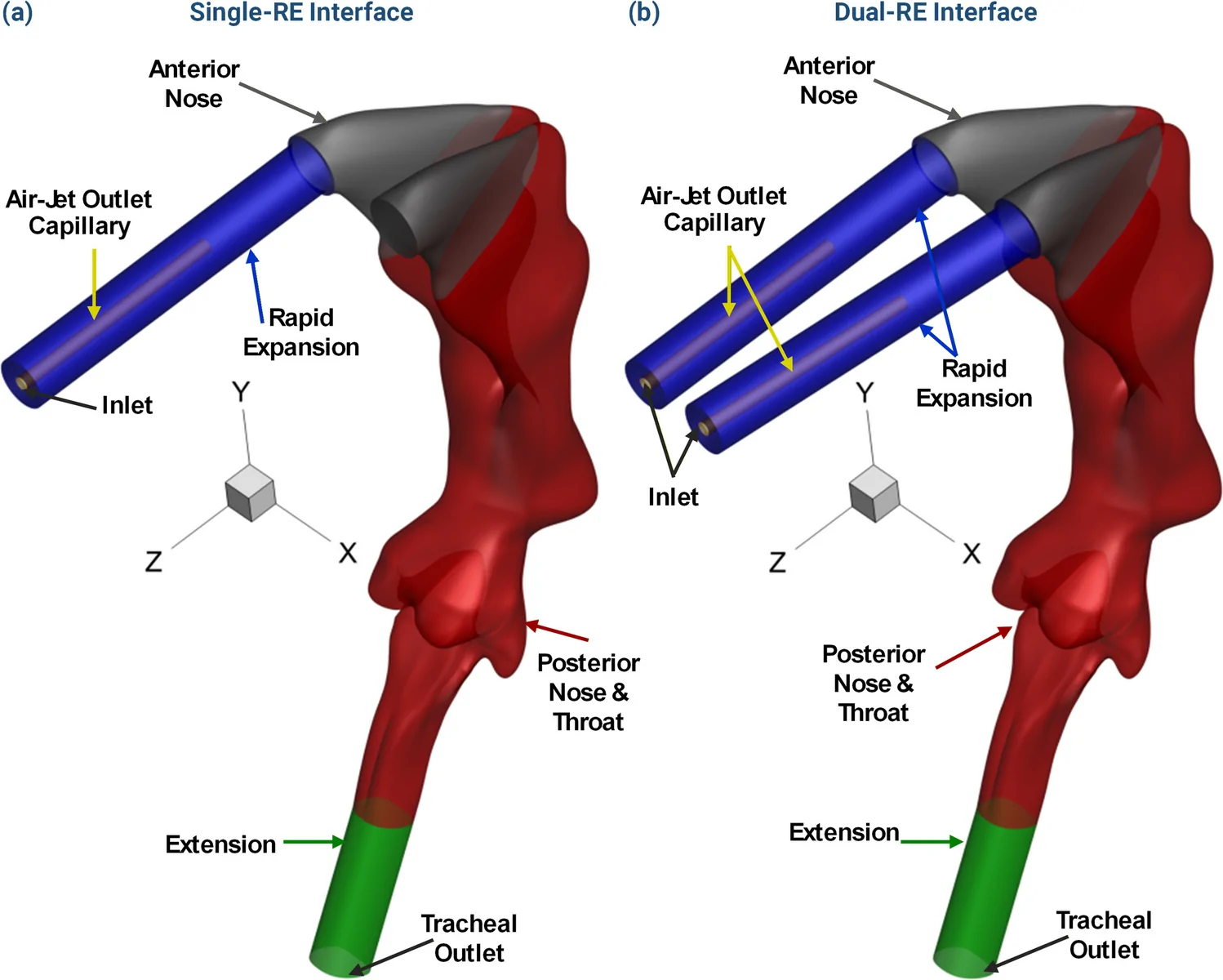
CFD domains (interior fluid space) for reference D2I transnasal aerosol delivery systems with different patient interfaces used in the *in vitro* trials and CFD model validation: (**a**) single-RE, and (**b**) dual-RE interfaces.

The computational model for D2I transnasal AST was constructed using two main geometric components: the PI and the preterm NT model (as shown in Fig. 3). The PI incorporated a diffusional flow path—specifically, a Rapid Expansion (RE) with a 4.24 mm internal diameter—that bridged the air-jet DPI device to the nasal prongs. Two fundamental PI architectures were studied: single-RE and dual-RE configurations. Note that minor design variations derived from these base models are introduced and detailed within the Results Section of the manuscript.

Since the air-jet DPI device itself was excluded from the CFD domain, its effect was replicated by including a segment of its outlet capillary (air-jet outlet capillary with a 0.89 mm diameter and a 17.5 mm length, as seen in Fig. 3a and b). The NT model comprised the anterior and posterior nasal passages, the throat, and the tracheal outlet, which was connected to a deposition filter. To ensure numerical stability, a numerical extrusion three times the diameter was attached to the tracheal outlet.

The CFD simulations were designed to mimic the flow dynamics of the *in vitro* model, each having a single inlet and a single outlet. For all transient simulations, a mass-flow inlet boundary condition was applied to the air-jet outlet capillary to enforce the time-dependent flow rate measured experimentally and shown in Fig. 2a. The associated turbulence at the inlet was specified by means of the hydraulic diameter of the inlet and the turbulent intensity (I), which was estimated by the following empirical equation dependent on the Reynolds number (Re) [61]:

2 $$I=0.16{\left(Re\right)}^{-{~}^{1}\!\left/ \!{~}_{8}\right.}$$

Conversely, steady-state simulations utilized a velocity inlet boundary condition at the air-jet outlet capillary. The inlet profiles of individual velocity components and turbulence parameters (k and ω) were prescribed using a fully developed flow profile. This profile was pre-calculated in a separate CFD analysis of a 3.24 L/min flow rate (the Q90 value, representing the 90th percentile of the time-resolved flow measurements shown in Fig. 2a) through an elongated pipe (length 30 times the diameter) with identical mesh density. All cases employed a pressure outlet boundary condition set to atmospheric pressure at the filter connection outlet.

For the discrete phase model (DPM), particles impacting the metal outlet capillary wall were set to reflect, based on experimental data showing zero deposition within the capillaries due to high shear stress on the smooth surfaces. All other flow domain walls, specifically those in the NT-region, were configured to trap particles upon impaction, and a uniform wall roughness of 50 µm was applied, consistent with the recommendation by Bass, *et al*. [43].

#### Meshing Approaches and Resolution

Meshing of all computational domains was performed by using the Meshing application within Ansys Fluent v2024R2 (Ansys Inc., Canonsburg, PA, USA). To adhere to the established best practices of our research group and capitalize on the advantages demonstrated in prior studies involving comparable geometries [46, 62, 63], the domains were meshed using an unstructured polyhedral cell topology supplemented by prismatic cell layers in the near-wall region. Optimal near-wall flow field resolution was ensured by maintaining the wall ${y}^{+}$ value near 1 across the entire domain (with the area-averaged wall ${y}^{+}$ consistently remaining below 1 for all faces). The prism cell layer in the near-wall region consisted of five cells and was constructed using a growth ratio of 1.2. The overall quality of the grid was ensured by enforcing a surface mesh skewness metric below 0.4 and verifying that the volume mesh exhibited an orthogonal quality greater than 0.25.

Rigorous mesh independence studies were conducted previously [41, 47] on analogous assemblies, including both the NT model and the single-RE/dual-RE PI variants, to define the optimal grid resolution. This investigation involved systematically comparing the volume-averaged velocity magnitude, turbulent kinetic energy (TKE), and deposition fraction (DF) across several mesh resolutions. The exact spatial resolution determined from these previous models was strictly preserved and implemented across all models investigated in the present study.

#### Numerical Models and CFD-Solver Settings

The CFD simulations were conducted within the commercial package Ansys Fluent v2024R2 (Ansys Inc., Canonsburg, PA, USA). The fundamental numerical methods, solver settings, and modeling approaches used in this work are adopted directly from a previous detailed publication by our group [47]. This prior study established an effective and efficient approach for turbulence modeling, which was based on the Low Reynolds Number (LRN) k-ω SST model, and was able to achieve accurate predictions of pharmaceutical aerosol deposition across a spectrum of turbulence regimes. Key recommendations from that work included modifications to the Eddy Interaction Model (EIM) and appropriate near-wall corrections.

The simulations were executed on a double-precision pressure-based 3D solver. Second-order spatial discretization schemes were employed for all transport equations, with gradient calculations performed using the Green-Gauss Node-based method. PREssure STaggering Option (PRESTO!) was chosen as the pressure interpolation scheme and the pressure–velocity coupling was achieved through the SIMPLEC scheme.

Steady-state simulations were considered to have converged when the residuals decreased by a minimum of four orders of magnitude and the volume-averaged velocity magnitude and TKE across the entire domain stabilized. Upon achieving a converged flow field, particles were introduced into the domain at the inlet face. The initial particle velocities were determined by their spatial location, adhering to the turbulent blunt velocity profile described by the 1/7th power law [64, 65]. The initial spatial distribution also followed a weighting that complies with the blunt profile of the fully developed turbulent flow. The injection file comprised polydisperse aerosol with a size distribution that replicated the experimentally determined PSDs (Fig. 2b) for the case. Specifically, the initially injected sizes were distributed across nine diameter size bins (0.206, 0.533, 0.874, 1.498, 2.578, 4.218, 7.297, 11.632, and 13.850 μm), with 3,000 particles per bin. Therefore, each steady-state simulation involved tracking a total of 27,000 parcels until they either deposited on a wall surface or exited the computational domain.

The majority of the results presented in this study were derived from transient runs, as these simulated the entire course of the actuation waveform detailed in the subsequent subsection. A second-order implicit temporal scheme was selected for these transient runs, using a fixed time step size of 0.0025 s. The particle injection method was similar to the steady-state approach but implemented in a time-dependent manner that replicated the experimentally determined emission profile of the aerosol, where the mass injected at each time step was varied to satisfy the recorded profile (Fig. 2b). The total number of injected particles varied slightly across cases, ranging approximately from 520,000 to 580,000. If any fraction of the injected particles remained suspended at the end of the actuation, it was included in the reported depositional losses by numerically pushing them to the nearest wall at the final time step. This choice was based on the assumption that these particles would likely deposit within the upper airway or be exhaled rather than contributing to the lung transmission. Since the exhalation phase was not modeled, our treatment of suspended particles by classifying them as regional losses serves as a conservative safeguard against overestimating lung delivery.

To quantify regional particle deposition, the deposition fraction (DF) was calculated. DF for any region is defined as the ratio of particle mass deposited in that region to the total particle mass released from the outlet capillary of the aerosolization chamber, expressed as a percentage:

3 $$DF_r=\frac{Particle\;mass\;deposited\;in\;region\;r}{Particle\;mass\;released\;from\;the\;aerosol\;outlet\;capillary}\times100\%$$

Particles exiting the computational domain (which essentially deposit on the tracheal filter of the *in vitro* setup) were quantified using the same metric and defined as the tracheal filter DF:

4 $$DF_{TF}=\frac{Particle\;mass\;deposited\;in\;the\;tracheal\;filter}{Particle\;mass\;released\;from\;the\;aerosol\;outlet\;capillary}\times100\%$$

This metric $D{F}_{TF}$ is interchangeably referred to as the *Lung Transmission Efficiency*. Consequently, the sum of all calculated DFs (including the tracheal filter DF) totals 100%. In addition to DF, which normalizes deposition to the total emitted dose, the Deposition Efficiency (DE) was calculated to evaluate the local retention characteristics of specific segments of the system. The DE of any particular region was defined as the ratio of the particle mass deposited to the particle mass entering that region, expressed as a percentage:

5 $$DE_r=\frac{Particle\;mass\;deposited\;in\;region\;r}{Particle\;mass\;that\;entered\;region\;r}\times100\%$$

## Results and Discussion

### *In Vitro *Testing Data of Preliminary Designs and CFD Validation

The *in vitro* testing results of the D2I transnasal aerosol delivery systems, using both single-RE and dual-RE interface designs, are summarized in Table 1. The reported values represent the mean mass percentages of the nominal formulation loaded dose along with the standard deviations (SD) derived from the experimental replicates (*n*= 3).

**Table 1:** Summary of the deposition fractions* measured during all *in vitro* trials and those predicted by the CFD simulations in this study for transnasal aerosol delivery to the preterm infant nose-throat model at a target peak flow rate of 3 L/min

Location of deposition	Single-RE		Dual-RE	
	*In vitro*	CFD**	*In vitro*	CFD**
**Device (%)**	23.8 (2.3)	N/A	28.0 (1.5)	N/A
**Patient Interface (%)**	14.4 (0.9)	17.9	14.3 (4.4)	13.9
**Nose & Throat (%)**	32.9 (7.2)	29.7	24.9 (2.2)	20.3
**Tracheal Filter (%)**	28.9 (1.5)	28.5	32.9 (2.8)	37.7

*Values are mean (SD) formulation mass expressed as a percentage (*n*=3) of the device loaded dose

**CFD predictions were adjusted by the experimental emitted fraction to represent a percentage of the loaded dose

The use of the single-RE and dual-RE configurations of the iDP-ADS resulted in slightly different initial aerosol PSDs emitted from the aerosolization chamber (see Fig. 2b). The mass median aerodynamic diameter (MMAD) was 1.74 ± 0.02 μm for the dual-RE interface and 1.59 ± 0.01 μm for the single-RE interface. The reported values represent the corresponding average value ± SD derived from the experimental replicates (n = 3). Examination of the regional deposition fractions revealed that the NT region was the primary site of aerosol loss with significant differences between designs, whereas depositional losses within the interface components were comparable. Notably, the single-RE interface resulted in a higher NT deposition (32.9%) compared to the dual-RE design (24.9%). This difference is attributable to the fluid dynamics of the inlet air-jet, which in the case of the single-RE variant retains higher momentum and turbulence intensity, thereby increasing the likelihood of aerosol particles depositing on the nasal cavity surfaces. In contrast, the dual-RE design divides the flow, reducing the momentum of individual jets entering the nostrils. This explanation is consistent with the concept that inertial impaction is governed by the Stokes number (Stk), which is proportional to fluid velocity (u) and the square of the particle diameter (${d}_{p}$). A simplified comparison of the respective Stokes numbers of both configurations, using the MMAD as the characteristic particle diameter, demonstrates that the Stk is 1.67 times higher in the single-RE configuration than in the dual-RE variant:

6 $$\frac{{Stk}_{Single}}{{Stk}_{Dual}}={\left(\frac{{d}_{p,single}}{{d}_{p, dual}}\right)}^{2}\times \frac{{u}_{single}}{{u}_{dual}}={\left(\frac{1.59 \mu m }{1.74 \mu m}\right)}^{2}\times \frac{2{u}_{dual}}{{u}_{dual}}=1.67$$

This demonstrates that the aerodynamic advantage of a smaller particle diameter in the single-RE variant—despite its squared influence on impaction—was outweighed by the disadvantage caused by the doubling of the fluid velocity. Consequently, the lung transmission efficiency, quantified by the mass percentage collected on the tracheal filter, was larger for the dual-RE interface (32.9%) compared to the single-RE variant (28.9%).

**Fig. 4:**
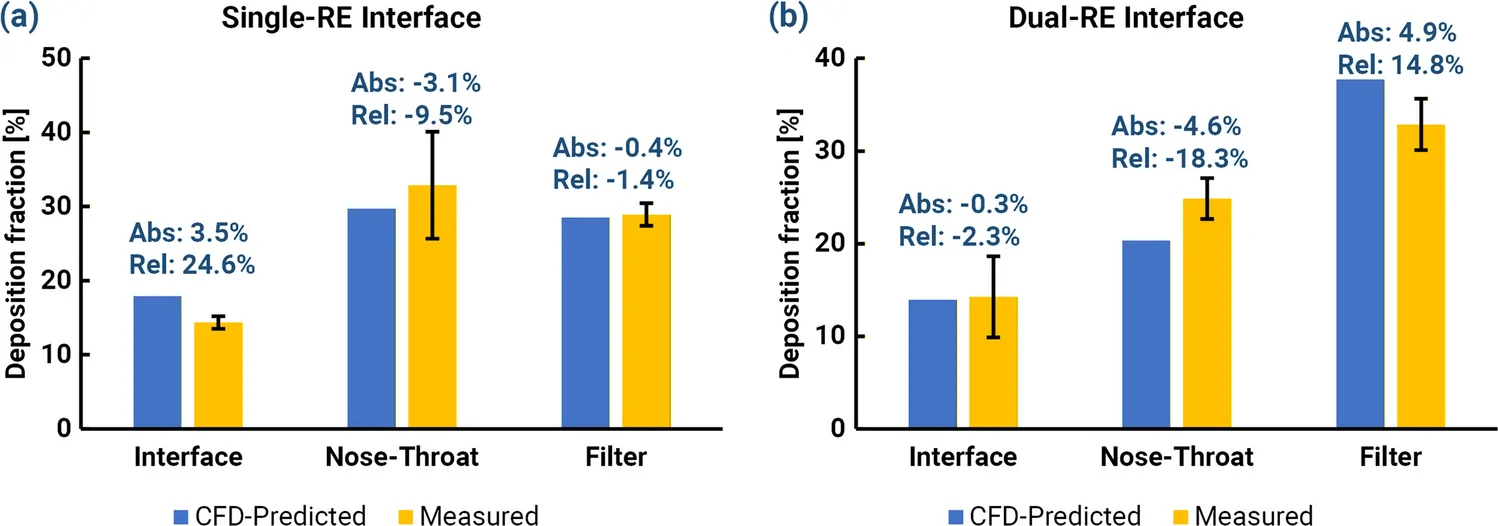
Comparison between the experimentally measured and CFD-predicted deposition fractions in two systems for the transnasal delivery of DPI-generated aerosol at a target peak flow rate of 3 L/min to a preterm infant nose-throat model; the different systems are comprised of different patient interfaces: (**a**) single-RE interface, (**b**) dual-RE interface. (Abs: absolute difference between the means, rel: relative difference between the means).

A direct comparison of the regional DFs predicted by the CFD model against the measured *in vitro* data is illustrated in Fig. 4 and summarized in Table 1. It is noted that the computational domains excluded the internal geometry of the DPI device or the aerosolization chamber; therefore, device emptying fractions were not predicted. To ensure a rigorous comparison, the CFD predictions were adjusted to account for the experimentally observed mean chamber retention for each interface type. Overall, the numerical predictions showed alignment with the experimental measurements for both interface configurations regarding trend and approximate magnitude. The maximum and mean relative differences were 24.6% and 11.7%, respectively, while the maximum and mean absolute differences were 4.9% and 2.8%, respectively.

For the single-RE interface (Fig. 4a), the absolute difference between measured and predicted DFs was consistently low, ranging from 0.4% to 3.5% across all regions. The model demonstrated high accuracy in predicting filter deposition, with an absolute difference of only −0.4% and a relative difference of −1.4%. However, a higher relative difference (24.6%) was observed in the interface region, where the CFD model predicted higher deposition (17.9%) than the experiment (14.4%). This prediction is deemed physically plausible and likely more representative of the aerodynamic limitations of the design. Specifically, the single-RE interface generates an inlet jet with approximately twice the momentum of the bifurcated branches of the dual-RE interface. The CFD model captured the enhanced inertial impaction and turbulent dispersion losses inherent to this high-momentum flow, whereas the experimental data—showing nearly identical losses for both single-RE and dual-RE interfaces (approx. 14%)—may have masked this effect. Despite this relative deviation, the absolute difference remains small (3.5%), and the model correctly identifies the trend of lower lung transmission for this design. This finding aligns with our previously reported data where single-path interfaces consistently resulted in elevated interface as well as anterior nasal deposition and reduced lung transmission compared to bifurcated systems [41, 47].

Regarding the dual-RE interface (Fig. 4b), the CFD model exhibited excellent agreement in the interface region, yielding an absolute difference of −0.3% and a relative difference of −2.3%. The most notable discrepancy occurred at the filter, where the simulation overpredicted deposition by an absolute value of 4.9% (relative difference of 14.8%). This overprediction corresponds with an underprediction of NT deposition (absolute difference −4.6%), implying that a fraction of particles predicted to pass through the NT region were captured there during experiments. This deviation is likely attributable to necessary model simplifications, such as the exclusion of particle–particle cohesion, agglomeration, and electrostatic effects. These physical phenomena, which are known to enhance depositional losses in complex geometries like the nasal cavity, would have a more pronounced impact in the dual-RE system where the split flow creates regions of lower velocity and longer residence time for the particles compared to the high-velocity single-RE system.

Despite these modest discrepancies, the predicted regional trends were consistent with measurements for both geometries. It was shown that the CFD framework reliably resolved the aerosol transport behavior, with deviations largely remaining within acceptable accuracy targets for extrathoracic deposition modeling (< 20% relative error for the critical lung dose).

### Importance of Polydisperse Aerosol Size and Transient Simulations

**Fig. 5:**
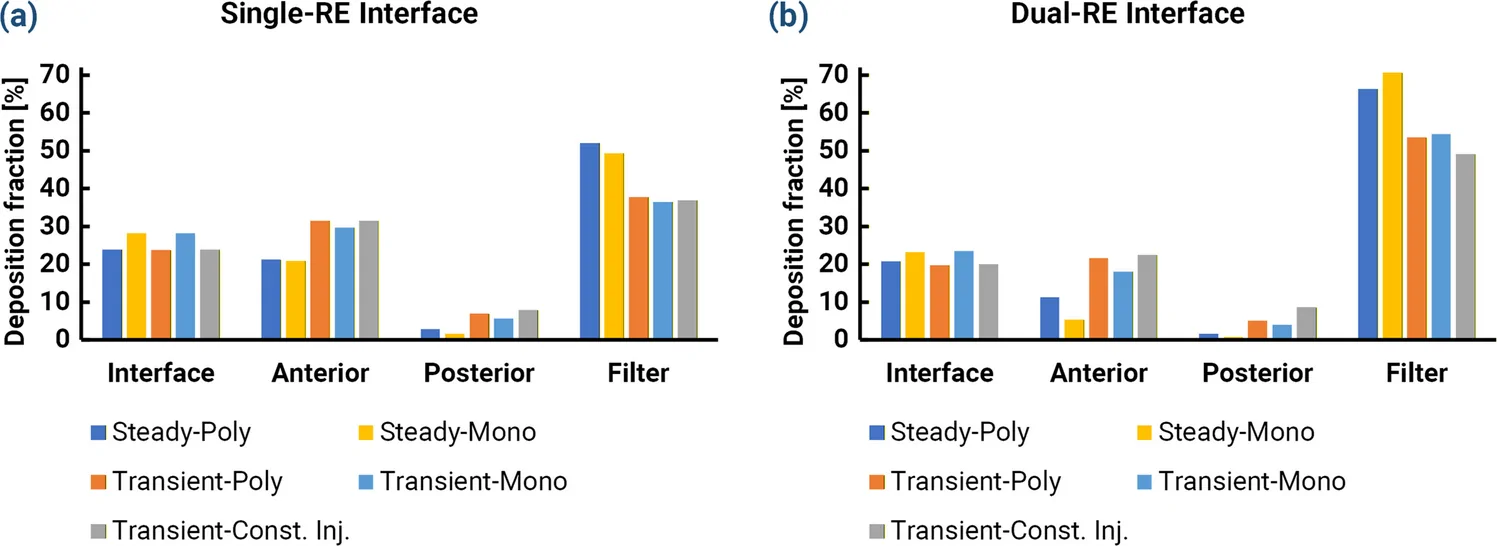
The effect of employing polydisperse aerosols and a fully transient approach during CFD simulations, shown by a direct comparison of the predicted local particle deposition patterns in various regions of the D2I transnasal aerosol delivery systems operated at a target peak flow rate of 3 L/min with different interfaces: (**a**) single-RE, and (**b**) dual-RE interfaces.

Using the validated CFD model, modeling assumptions were further investigated. To delineate the individual contributions of modeling assumptions related to aerosol size distribution and flow unsteadiness, a systematic sensitivity analysis was conducted. Simulations were performed using both polydisperse and monodisperse (set to the MMAD of 1.59 μm and 1.74 μm for the single-RE and dual-RE variants, respectively) initial PSD under both steady-state and transient flow conditions. These investigations were carried out for both the single-RE and dual-RE interface variants to determine if the observed effects were geometry-dependent. The resulting regional deposition fractions for the single-RE and dual-RE interfaces are visualized in Fig. 5a and b, respectively.

#### Effect of Initial Particle Size Distribution

For both geometries, approximating the aerosol as a monodisperse population based solely on its MMAD (represented by Steady-Mono in Fig. 5) resulted in a distinct shift in deposition patterns compared to the realistic polydisperse case (denoted by Steady-Poly in Fig. 5), which was run with a distribution of sizes based on the experimentally measured PSD (Fig. 2b). This divergence confirms that the nonlinear size dependence of local deposition mechanisms cannot be fully captured by a single average particle diameter. While this qualitative outcome of encountering different deposition fractions was anticipated, the current analysis identified the exact nature of the shift in deposition and explicitly quantified the magnitude of the difference. For instance, in the dual-RE interface under steady-state conditions, the monodisperse assumption led to an 11.5% (relative difference) overprediction of deposition within the interface compared with the steady-polydisperse case (23.2% vs. 20.8%, respectively) and a substantial underprediction of losses in the NT region (6.2% vs. 12.9%, respectively). This behavior is consistent with the enhanced turbulent dispersion of monodisperse aerosols due to their relatively small size. As a result, increased deposition losses were observed for the monodisperse aerosol compared with the polydisperse aerosol in the jet-induced high-turbulence region within the interface. Conversely, lacking the larger particle fraction present in the polydisperse distribution led to decreased inertial impaction in the nasal cavity. Consequently, a greater fraction of the finer particles remained entrained in the core flow and was transported deeper into the system with Steady-Mono, ultimately causing the monodisperse assumption to yield an overprediction of the lung transmission (filter deposition) of approximately 4.3% compared with the steady-polydisperse case (relative difference of 6.5%). Similar trends were observed for the single-RE interface (Fig. 5a) as well as in the corresponding transient runs, confirming that accurate modeling of the full polydisperse spread is essential to capture the combined effect of the resulting deposition mechanisms of turbulent dispersion and inertial impaction.

#### Impact of Transient Flow Physics

The comparison between steady-state and transient simulations revealed that time-dependent flow physics are also critical for accurate prediction of lung transmission in a D2I transnasal AST platform. The steady-state simulations, based on the Q90 flow rate (3.24 L/min), significantly overpredicted filter deposition compared to the full transient runs (Transient-Poly in Fig. 5). For the dual-RE interface, the steady-state approach predicted a filter deposition of 66.3%, whereas the transient simulation predicted 53.5%, a substantial relative difference of roughly 24%. This discrepancy was driven largely by a marked underprediction of NT deposition in the steady case (12.9%) compared to the transient case (26.7%). This divergence is attributable to the differences in particle dispersion behavior. The constant high-velocity flow in the steady-state approximation forces particles to follow the streamlines more closely, minimizing residence time and opportunities for dispersion. In contrast, the transient simulations accurately captured the variable velocity profile of the actuation, which directly impacted the instantaneous momentum and followed path of the particles. Analysis of the particle tracks revealed that the average residence time of deposited particles in the steady-state cases was approximately 40% lower than that predicted in the transient cases (e.g., 12.6 ms vs. 7.3 ms for the dual-RE interface and 5.76 ms vs. 3.82 ms for the single-RE interface). Hence, it was concluded that the variable velocity field in the transient simulations disperses particles more realistically, making them more susceptible to deposition mechanisms such as turbulent dispersion and sedimentation, particularly in the NT region.

A fully transient simulation requires not only employing a time-dependent fluid flow profile but also a variable aerosol mass flow rate (emission profile). Therefore, the cases run under a transient flow field but using a constant mass injection rate (Transient-Const. Inj. in Fig. 5) instead of the experimentally measured aerosol emission profile helped isolate the influence of the injection dynamics. While the interface losses remained comparable, the constant injection method noticeably overpredicted NT deposition compared with transient injection (31.1% vs. 26.7% for dual-RE, respectively) and consequently underpredicted lung transmission (49.1% vs. 53.5%, respectively). This error arose because particles injected towards the end of the actuation in the constant rate cases did not have sufficient time to clear the domain and thus became trapped in the nasal cavity as the device flow decelerated and eventually ceased. Moreover, the uniform injection rate exposed a larger proportion of the total particle mass to low-momentum flow during the ramp-up and ramp-down phases of the actuation, making them prone to escaping the core flow. Conversely, the realistic emission profile varied the aerosol mass consistent with the flow rate and concentrated the particle mass earlier in the actuation course, allowing for better clearance and transmission. Therefore, it was concluded that a realistic emission profile is needed in conjunction with transient flow rates.

### Impact of Upstream Air Volume

**Fig. 6:**
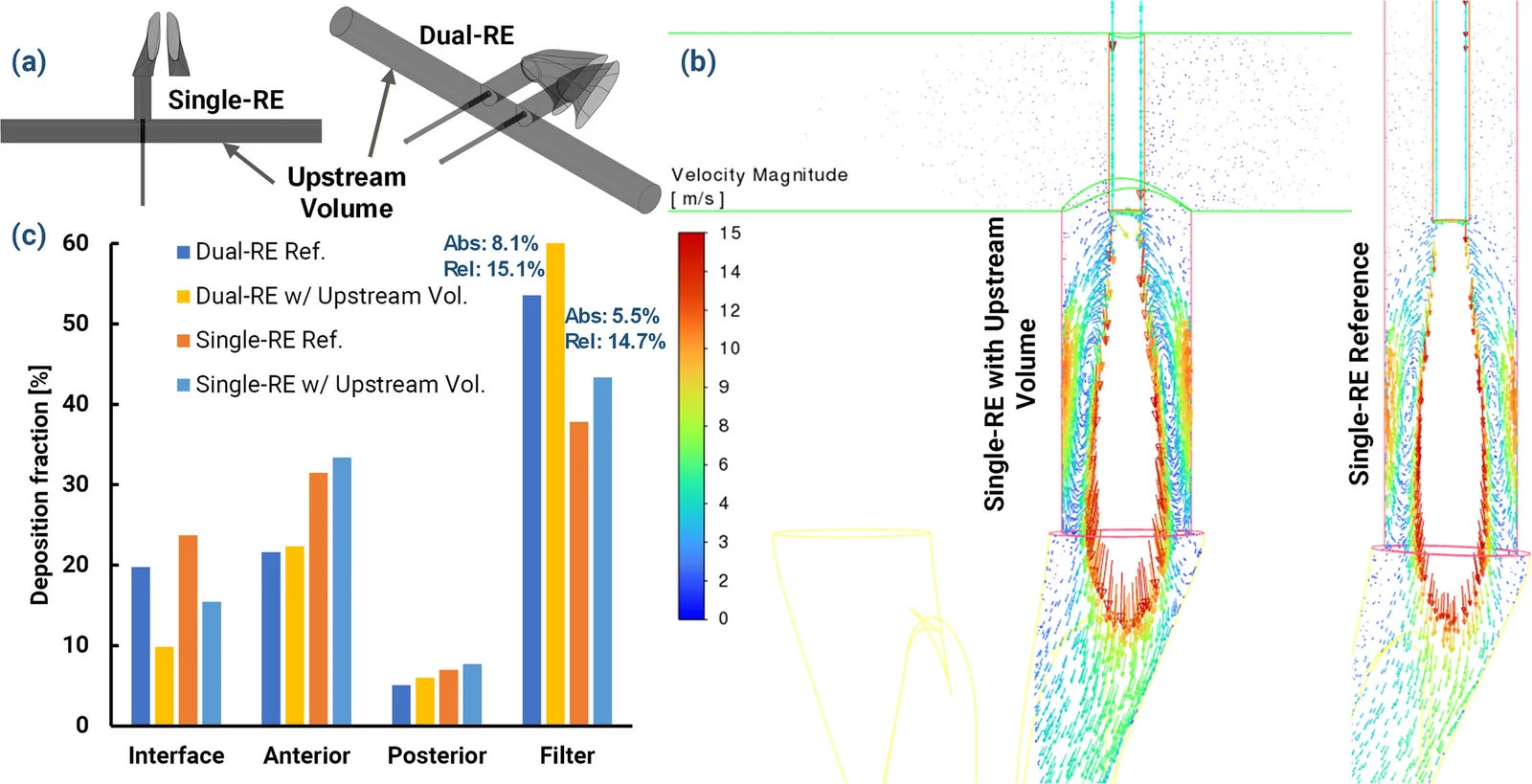
(**a**) The modified interface incorporating an upstream volume for both the single-RE and dual-RE interfaces. (**b**) The effect of the upstream volume on the flow field, visualized by the CFD-predicted velocity vectors in a single-RE interface compared directly with the base/reference variant at a flow rate of 3 L/min. (**c**) Direct comparison of the performance of single-RE and dual-RE interfaces (with and without an upstream volume) based on the predicted local particle deposition patterns in various regions of the transnasal aerosol delivery systems operated at a target peak flow rate of 3 L/min.

Following the investigation of specific modeling approaches and simplifications, the focus of this study shifted to interface design aspects. The primary objective of this section was to investigate the influence of an upstream air volume—specifically, the region upstream of the DPI outlet capillary jet—on flow-field development and subsequent aerosol transport in D2I transnasal delivery. To evaluate this, a perpendicular air volume (~ 1.8 mL) was added just upstream of the single- and bifurcating-path prong designs as pictured in Fig. 6a. The specific volume and perpendicular geometry were extracted from a standard nCPAP interface (Hudson RCI), replicating the geometry of its CPAP line. While the upstream volume was the theoretical variable of interest, utilizing the nCPAP interface offered potentially significant practical advantages. Because nCPAP support is routinely applied to infants with respiratory distress [18, 66], an integrated design enables “on-demand” aerosol delivery to be realized without requiring an interface exchange. By utilizing the existing nCPAP geometry as the upstream volume component, the transnasal AST could be administered whenever required. This would simply require an integrated valve system to regulate flow through the inspiratory and expiratory limbs of the CPAP line.

In the realized design (Fig. 6a), the outlet capillary of the air-jet DPI penetrates the cylindrical air volume space and extends directly into the nasal prongs. To quantify the effect of this upstream volume, a sensitivity analysis was conducted by comparing the performance of both single-RE and dual-RE geometries against their respective reference variants with no upstream air volume.

CFD simulations revealed that the upstream air volume significantly altered the expansion dynamics of the inlet jet. As the high-velocity aerosol jet exits the capillary and rapidly expands into the interface’s diffusion element, a localized zone of negative pressure forms around and upstream of the jet (illustrated by velocity vectors in Fig. 6b). This pressure gradient induces flow separation and elevated turbulence levels, which typically promote particle deposition through impaction and turbulent dispersion. In the reference configurations, the backflow region did not extend substantially upstream into the space around the capillary overhang. However, the presence of the larger upstream volume allowed the negative-pressure region to extend fully into the cavity of the capped air volume space. This extension enhanced the recirculation strength, drawing additional air from the core flow. As visualized in Fig. 6b, this upstream entrainment produced a sustained inward return flow as well. Combined with the strengthened recirculation, this returning flow acted as a hydrodynamic “shield” or protective shear layer along the walls in the vicinity of initial jet expansion, effectively keeping particles away from the interface surfaces and thereby reducing wall deposition.

Figure 6c presents a direct comparison of the local deposition fractions in both interface types, with and without the upstream air volume. Quantitative analysis confirmed that the shielding effect substantially reduced interface losses. For the dual-RE geometry, interface deposition decreased from 19.8% to 9.8% (a 50.2% reduction). Similarly, losses in the single-RE interface dropped from 23.7% to 15.5% (a 34.9% reduction).

The reduction in upstream losses increased the mass of aerosol available for downstream transport. Although deposition in the NT region increased slightly—rising from 26.7% to 28.3% for the dual-RE variant and from 38.4% to 41.1% for the single-RE variant—this increase was attributable to the greater powder mass flux entering the nasal cavity rather than to any significantly adverse change in local deposition efficiency. Ultimately, the inclusion of the upstream volume substantially improved lung transmission efficiency. Filter deposition for the dual-RE design increased from 53.5% to 61.6% (Fig. 6c), with the single-RE design showing a comparable improvement (38.4% to 43.4%).

These findings confirm that upstream air volume plays a constructive role in shaping the flow field, mitigating particle wall deposition, and improving lung delivery efficiency. The consistency of the observed behavior across both geometries underscores the robustness of this mechanism.

### Impact of Interface Length and Turbulent Dispersion

**Fig. 7:**
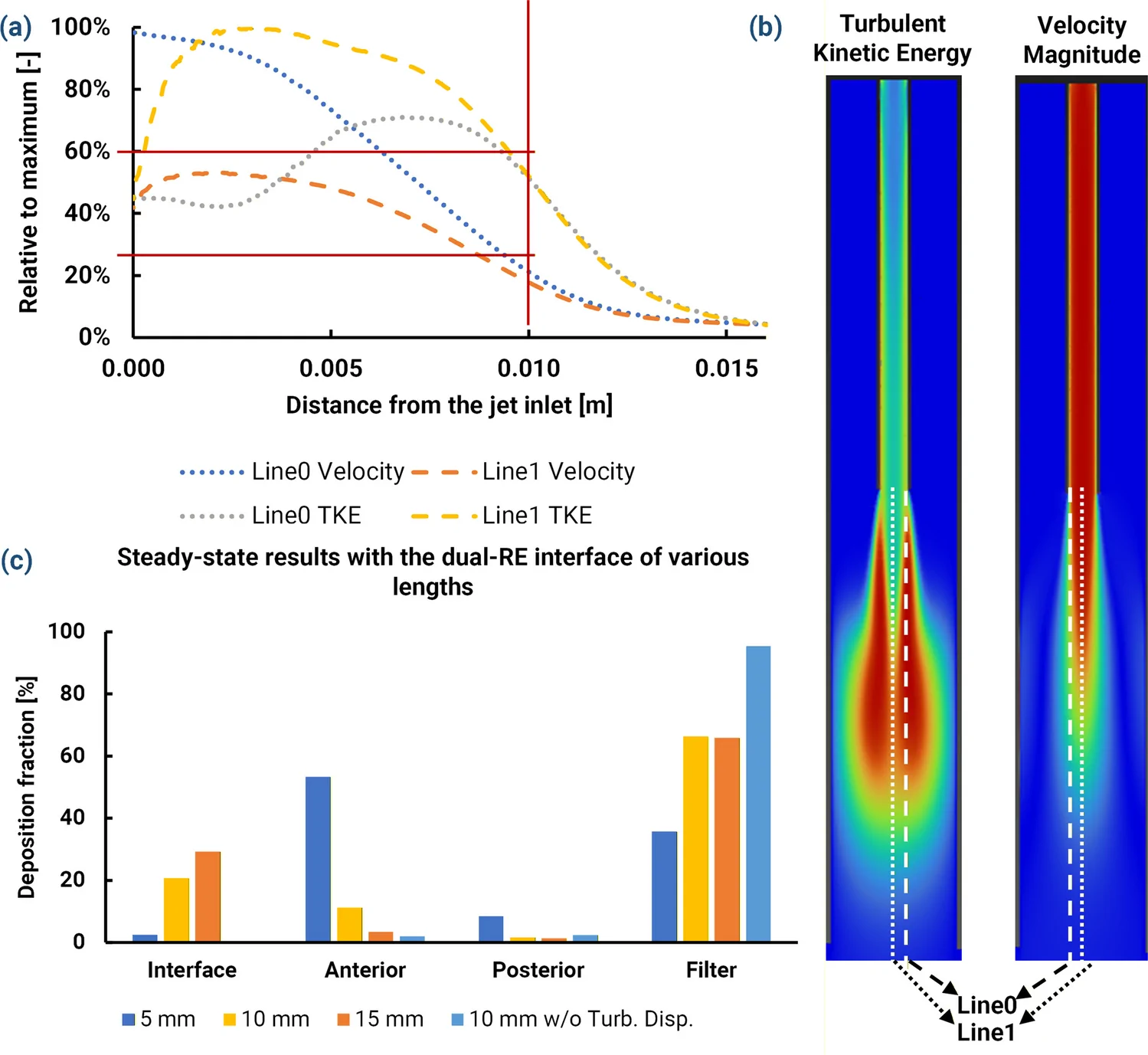
(**a**) Spatial profiles of velocity and turbulent kinetic energy (TKE) for different interface lengths. (**b**) Contour plots of velocity and TKE in the interface, demonstrating the dissipation of the jet. (**c**) Impact of the interface length on delivery performance, visualized by the CFD-predicted local deposition fractions in various regions of the transnasal aerosol delivery system simulated at a constant flow rate of 3.24 L/min (Q90 of the target peak flow rate of 3 L/min) with the dual-RE configuration for various interface lengths.

Having established the role of the upstream volume in the interface design, the next phase of the study focused on the internal dimensions of the interface—specifically, the length of the rapid expansion region. Varying this length directly influences the degree of turbulence introduced into the nasal cavity. Hence, this section systematically investigates the impact of the RE length on aerosol deposition and delivery performance, alongside the contribution of another modeling parameter, i.e., turbulent dispersion in particle transport. A sensitivity analysis was conducted to optimize the length of the RE component, with the objective of understanding how the diffusional pathway length influences dissipation of the high-velocity inlet jet prior to entering the nasal cavity. Three interface lengths were evaluated: 5 mm, 10 mm, and 15 mm. As illustrated by the white dotted/dashed lines in Fig. 7b, this length is defined as the exact distance between the aerosol capillary outlet and the end of the nasal prong. To isolate the specific contribution of turbulence, an additional simulation was performed for the 10 mm length with turbulent dispersion of particles deactivated. It is noted that while this neglect of turbulent dispersion is a theoretical abstraction, it was utilized solely to elucidate the role of turbulence in the CFD predictions. Results for the dual-RE geometry are presented here, as single-RE simulations exhibited qualitatively similar trends.

Given the increased computational cost associated with fully transient simulations, steady-state simulations were employed, since the primary objective was to identify relative trends and differences rather than high-fidelity absolute deposition fractions. Moreover, the contribution of transient effects has already been examined in a preceding section. All cases were simulated at the Q90 flow rate (3.24 L/min), which was previously shown by our group [43, 46, 47, 62] to be most representative of the transient DPI actuation profile. Q90 is defined as the volumetric flow rate below which 90% of the rapidly sampled flow rate measurements reside.

To rationalize the observed deposition patterns, the fluid dynamic development of the air-jet was first analyzed. Figure 7a presents the profiles of velocity magnitude and turbulent kinetic energy (TKE) along the centerline (Line 0) and along a line extending from the capillary wall (Line 1), where TKE was observed to peak. Figure 7b provides the corresponding contour plots for the 15 mm interface length. The discrepancy between Line 0 and Line 1 serves as an indicator of the degree of jet dissipation.

At a length of 5 mm, the jet remained highly focused; the discrepancy between the centerline and Line 1 was substantial, with TKE near its peak and the core velocity retaining approximately 90% of its maximum value. This behavior indicates pronounced non-uniformity across the interface width. At 10 mm, the profiles began to converge, with TKE and velocity dissipating to approximately 57% and 17% of their maximum values, respectively. By 15 mm, the line profiles coincided, indicating near-complete dissipation of the jet, with both velocity and TKE reduced to approximately 6% of their maximum values. These results indicate that extending the interface length beyond 15 mm offers negligible additional benefit with respect to jet dissipation.

These observations highlight the competing effects associated with increasing interface length: while longer expansions promote more uniform flow and enhanced jet dissipation, they also increase the internal surface area over which particles may deposit. Identifying an optimal length therefore requires balancing these opposing trends.

Figure 7c illustrates the predicted deposition fractions across the varying interface lengths and dispersion settings. The sensitivity case without turbulent dispersion clearly demonstrates the dominant role of turbulence-driven particle transport. When turbulent dispersion was disabled for the 10 mm interface, deposition within the interface was virtually eliminated (0.1%), compared to 20.8% when turbulence was included. A marked reduction in deposition within the NT region was also observed, decreasing from 12.9% to 4.5%, with the most pronounced reduction occurring in the anterior region, where deposition dropped from 11.3% to 2.0%. Consequently, lung transmission (filter deposition) increased from 66.3% to 95.4%. This difference indicates that turbulent dispersion accounts for an approximate 29.1% reduction in the delivered dose. Therefore, turbulence plays a profound role in particle–wall interactions for simulations conducted in this flow regime and cannot be neglected in accurate predictive modeling.

With turbulent dispersion enabled, increasing the interface length from 5 to 15 mm resulted in a nonlinear increase in deposition within the interface itself, rising from 2.5% at 5 mm to 20.8% at 10 mm, and further to 29.3% at 15 mm. Concurrently, deposition in the NT region decreased sharply, from 61.8% at 5 mm to 12.9% at 10 mm and to 4.8% at 15 mm. This trend is consistent with progressive jet dissipation upstream of the nasal cavity, which reduces both inertial impaction and turbulence-driven dispersion within the NT region.

The combined effect of these opposing trends is reflected in the lung transmission efficiency. Filter deposition increased substantially when the interface length was extended from 5 to 10 mm, rising from 35.7% to 66.3%. However, a further increase to 15 mm did not yield additional improvement, with filter deposition remaining essentially unchanged at 65.8%. Although local deposition patterns continued to shift—specifically, increased losses within the interface and reduced losses in the NT region—the overall delivery performance plateaued beyond 10 mm. This indicates that beyond a critical length, the penalty associated with increased interface deposition outweighs the benefit gained from reduced NT deposition.

From a design optimization perspective, the 10 mm rapid expansion length represents one logical compromise. Although TKE and outlet velocity remained elevated relative to the 15 mm case, the nasal cavity provides additional volume for further jet dissipation. It is therefore likely that both velocity and TKE were reduced to below 20% of their maximum values before the flow reached the posterior regions of the nasal airway when a 10 mm interface was used. Since extending the interface length beyond 10 mm provided no net gain in lung delivery efficiency while increasing device size, the 10 mm configuration may offer an advantageous balance between jet dissipation and minimization of interface loss.

However, from a clinical perspective, the 15 mm configuration may offer distinct advantages by shifting depositional losses from the patient to the device. Retaining a larger fraction of the lost dose within the removable interface reduces the mass impacting the nasal walls, thereby lowering the risk of airway obstruction. This is particularly important for surfactant administration, where high local concentrations can interact with mucosal moisture to form viscous coatings or froth that obstructs airflow. Therefore, while the 10 mm length maximizes delivery efficiency, the 15 mm length effectively keeps excess drug outside the nose, potentially improving patient tolerance. It should be noted, however, that the potential clinical risks associated with excessive off-target deposition in the upper airways remain speculative, as direct *in vivo* evidence for dry powder surfactant administration is currently lacking.

### Impact of Ergonomic Alignment of the Interfaces

Building on the findings of the previous section, where turbulent dispersion was identified as the dominant mechanism governing depositional loss within the interface and inertial impaction was shown to play an increasingly important role within the NT region, the alignment of the dual-RE interface relative to the nasal passage was investigated as a potential strategy to further improve delivery efficiency. It was hypothesized that aligning the prongs with the anatomical orientation of the nasal passage would streamline the jet outlet trajectory, thereby reducing impaction-driven losses in the NT region, particularly in the anterior part. This hypothesis is supported by our previous investigation into CPAP overlay interfaces [41], where optimizing prong angle reduced nasal deposition.

**Fig. 8:**
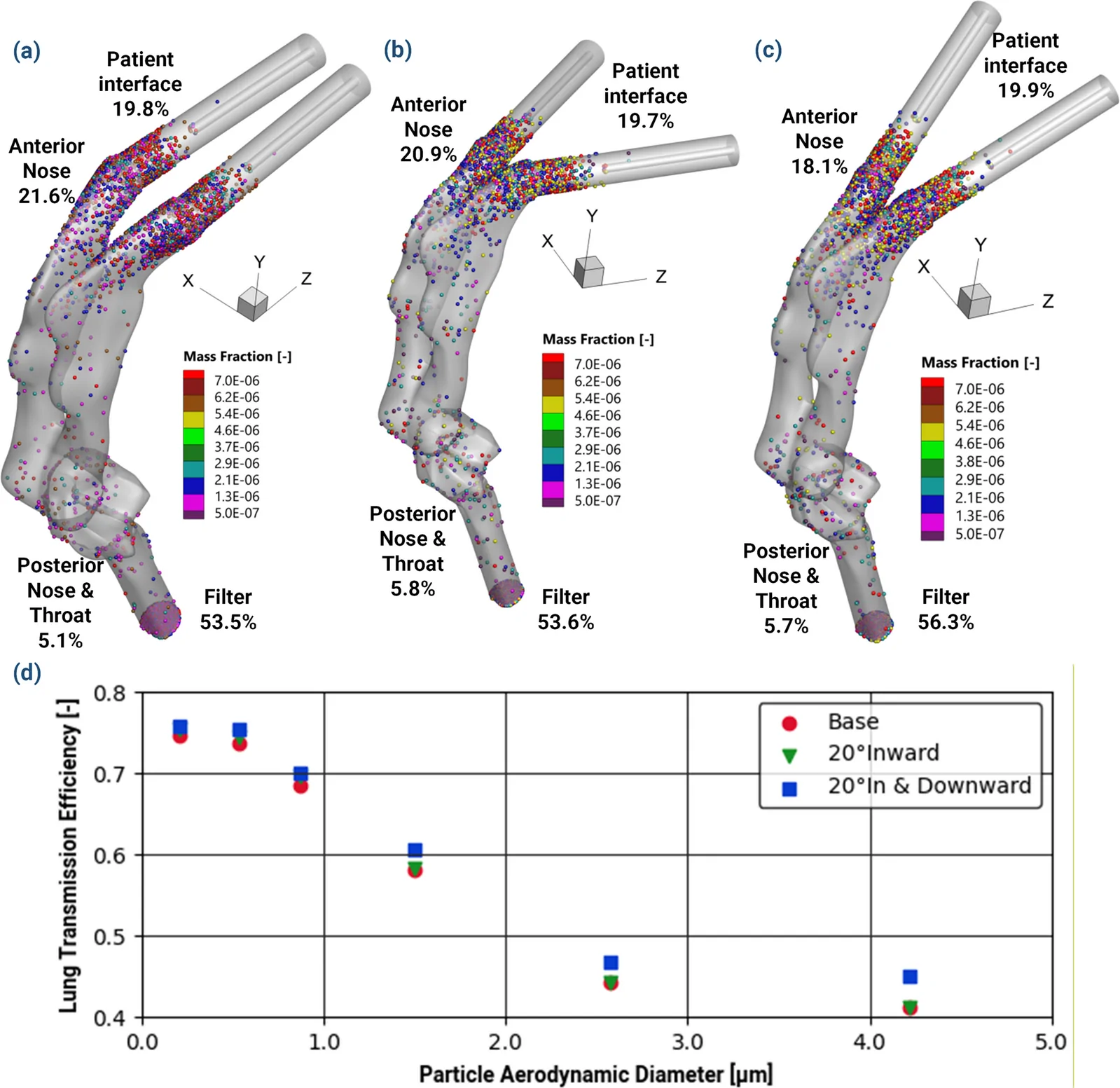
The effect of interface alignment on delivery performance, visualized by the CFD-predicted local deposition fractions in various regions of the transnasal aerosol delivery systems operated at a target peak flow rate of 3 L/min with different interfaces: (**a**) Initial base design of the dual-RE interface; (**b**) Dual-RE interface with a 20° inward angle; (**c**) Dual-RE interface with a 20° inward and 20° downward angle. (**d**) CFD-predicted lung transmission efficiency at a target peak flow rate of 3 L/min of different particle diameters for various interface alignments, demonstrating its differential impact on different sized particles.

To evaluate this effect in the context of the current D2I transnasal AST platform, two modified configurations of the dual-RE interface were developed. The first variant incorporated a 20° inward angulation of the prongs towards the septum (see Fig. 8b), while the second combined the 20° inward angle with a 20° downward angle relative to the interface's central axis, directing the inlet jets closer to the nasal floor (see Fig. 8c).

Figure 8a–c present a direct comparison of the regional deposition patterns for the investigated interfaces. Quantitatively, the 20° inward alignment alone resulted in only marginal changes. Interface deposition remained approximately 19.7–19.8%, while anterior nasal deposition decreased slightly from 21.6% in the baseline to 20.9%. Filter deposition showed a negligible increase from 53.5% to 53.6%. These modest differences indicate that inward angulation alone is insufficient to meaningfully alter the flow trajectory or reduce particle–wall interactions within the nasal passage.

In contrast, the addition of a downward orientation produced a detectable improvement in delivery performance. For the combined inward and downward alignment, filter deposition increased to 56.3%, representing an approximate 3% absolute increase relative to the baseline performance. This improvement was accompanied by a reduction in anterior nasal deposition to 18.1%, while interface deposition remained essentially unchanged. Deposition within the posterior nose and throat remained comparable across configurations (approximately 5.1–5.8%) as well, indicating that the observed gains were attributable primarily to reduced losses in the anterior nasal region.

To understand the differential impact of alignment on various particle sizes, lung transmission efficiency was examined by diameter class (see Fig. 8d). While only minor differences appeared between the baseline and inward-only configurations, the combined inward and downward alignment consistently improved transmission across all particle sizes. Notably, the magnitude of improvement increased with particle diameter, with the largest gains (approx. 6%) observed for the 4.22 μm particles. This trend is further indicative of reduced impaction losses, as larger particles with elevated Stokes number (Stk) are more susceptible to inertial deviation from the flow direction. These results support the conclusion that improved alignment primarily mitigates impaction-driven deposition rather than losses dominated by diffusion or turbulent dispersion.

Furthermore, beyond the demonstrated aerodynamic advantages, interface alignment carries clinical implications regarding patient comfort and potential safety. Therefore, a primary motivation for adopting the aligned geometry is improving ergonomic fit to maximize patient comfort. Importantly, our findings confirm that enhancing this ergonomic fit does not compromise aerosol delivery performance; rather, it offers a moderate enhancement in lung transmission efficiency.

### Impact of Front-Loading of Aerosol within the Supplied Breath

While interface geometry plays a critical role in deposition, the temporal characteristics of aerosol generation by the device are expected to be equally influential. In this section, the impact of front-loading of aerosol emission into the earlier time period of the delivered breath on deposition and delivery efficiency is investigated. In the context of the air-jet DPI device, front-loading refers to the specific functional capability of the device to aerosolize and emit the majority of the powder dose within the initial portion of the actuation cycle. For the device considered in this study, the total actuation duration is approximately 0.3 s; however, the design ensures that most of the powder is emitted within the initial 0.2 s (see Fig. 2). This reserves the final 0.1 s of the actuation airflow to serve as aerosol-free "chase air." This chase air is hypothesized to play a critical role in transporting emitted particles through the dead space of the interface, nasal passage, and upper airways toward the lower lung before the end of the inhalation breath.

To quantify the effect of front-loading, CFD-predicted deposition fractions were compared between two emission profiles for both the single-RE and dual-RE configurations. The first case employed the experimentally measured emission profile (shown in Fig. 2a), where the aerosol dose is emitted within approximately the first two-thirds of the actuation. The second case used a fabricated emission profile where the aerosol emission rate was directly proportional to the instantaneous flow rate, resulting in continuous emission until the flow decayed to zero.

Table 2 summarizes the predicted deposition fractions for these cases. A critical aspect of this analysis involved the treatment of suspended particles. The column titled "Final (pushed)" represents data where particles remaining suspended in the domain at the end of the simulation were computationally "pushed" to deposit on the nearest wall surface. The discrepancy between the standard and "pushed" datasets highlights the fraction of aerosols that fail to either deposit or clear the geometry by the end of the simulated flow-time. It should be noted that *in vivo*, a portion of these suspended particles would likely remain airborne and be exhaled rather than depositing entirely on the wall surfaces. However, analytically forcing this suspended fraction to the nearest walls in our model serves as a strictly conservative assumption with regard to depositional losses. By classifying these suspended particles as regional losses, we ensure they are rigorously excluded from the lung transmission fraction.

**Table 2:** Summary of the deposition fractions predicted by the CFD simulations of transnasal aerosol delivery to the preterm infant nasal model using the measured and flow-rate proportional aerosol emission profiles at a target peak flow rate of 3 L/min. The columns titled “Final (pushed)" contain data including suspended particles computationally deposited onto the nearest wall at the end of the simulation

Emission Profile	Single-RE			Dual-RE		
	Flow-rate proportional		Measured	Flow-rate proportional		Measured
**Deposition fraction**	Final	Final (pushed)	Final	Final	Final (pushed)	Final
**Interface (%)**	22.9	24.8	23.7	18.7	22.4	19.8
**Anterior (%)**	30.6	31.8	31.5	20.7	23.1	21.6
**Posterior (%)**	8.1	12.8	7.0	10.0	14.6	5.1
**Filter (%)**	30.5	30.6	37.8	39.8	39.9	53.5
**Suspended (%)**	7.9	N/A	N/A	10.8	N/A	N/A

The results clearly demonstrate the aerodynamic advantage of front-loading. With flow-rate proportional emission, a significant fraction of particles remained suspended in all regions at the end of the simulation (7.9% and 10.8% for the single-RE and dual-RE configurations, respectively). Transient solution animations (Supplementary Videos S1 and S2 for the single-RE and dual-RE configuration, respectively) visually capture this phenomenon, illustrating the delayed particle clearance associated with the proportional emission profile. Notably, the fraction of suspended particles increased in the downstream regions—being highest in the posterior nose and throat, moderate in the anterior nose, and lowest in the interface. For instance, in the dual-RE configuration, deposition in the posterior nose and throat rose from 10.0% in the final state to 14.6% in the pushed state, representing a 4.6% suspended fraction. Similarly, the anterior region showed an increase from 20.7% to 23.1%. In contrast, the interface region exhibited a smaller difference (18.7% final vs. 22.4% pushed). This increasing gradient of suspended particles from the interface to the posterior regions indicates that particles emitted late in the actuation cycle lacked sufficient residence time and flow momentum to traverse the dead space of the entire model.

Comparable trends were evident in the single-RE configuration as well. While overall suspension was lower than in the model with the dual-RE interface, the downstream bias persisted. The discrepancy between final and pushed deposition fractions was larger in the posterior region (8.1% vs. 12.8%) compared to the interface (22.9% vs. 24.8%). This confirms that even with the higher local velocities that are characteristic of the single-RE design, late-emitted particles are still prone to retention within the upper airways.

Conversely, the measured profile with front-loaded aerosol effectively eliminated this suspended fraction. Ultimately, shifting from a proportional to a front-loaded emission profile resulted in a substantial increase in lung delivery efficiency. Filter deposition increased by 7.2% for the single-RE variant and by 13.6% for the dual-RE variant. It is noteworthy that the gain was markedly larger for the dual-RE geometry. This difference is attributed to the dynamics of the continuous phase within the interface; as already explained, the dual-RE design splits the airflow, resulting in lower local velocities compared to the single-RE variant. Consequently, particles in the dual-RE system possess less velocity to clear the model and are more prone to suspension if not driven by early emission. In contrast, the single-RE variant, characterized by velocities roughly twice as high, inherently aids particle clearance, making the system slightly less reliant upon—though still significantly benefited by—front-loading.

In conclusion, it is important to design the aerosolization engine of the iDP-ADS such that the entire aerosol dose is emitted early in the actuation. This design requirement ensures sufficient time for transport to the lung and provides adequate chase air to drive particles to the lower respiratory region. Minimizing the suspended fraction is critical; practically, these particles result in either upper airway deposition or exhaled dose, wasting the therapeutic drug or increasing the risk of off-target deposition and subsequent systemic side-effects. Furthermore, this strategy is particularly vital for interfaces designed with lower peak flow rates or actuation volumes, where the momentum deficit makes front-loading a governing factor in achieving higher delivery efficiency.

### Impact of Device Actuation Flow Rate

The final parameter investigated for its influence on delivery performance was the device actuation flow rate of the iDP-ADS aerosolization engine. Flow rate is a critical governing factor, as it directly dictates the aerodynamic conditions—specifically inlet jet characteristics, secondary velocities, and shear stress—that drive powder bed fluidization, particle dispersion, and transport from the chamber. Consequently, both the temporal emission profile and the emitted particle size distribution (PSD) are intrinsically coupled to the actuation flow rate.

**Fig. 9:**
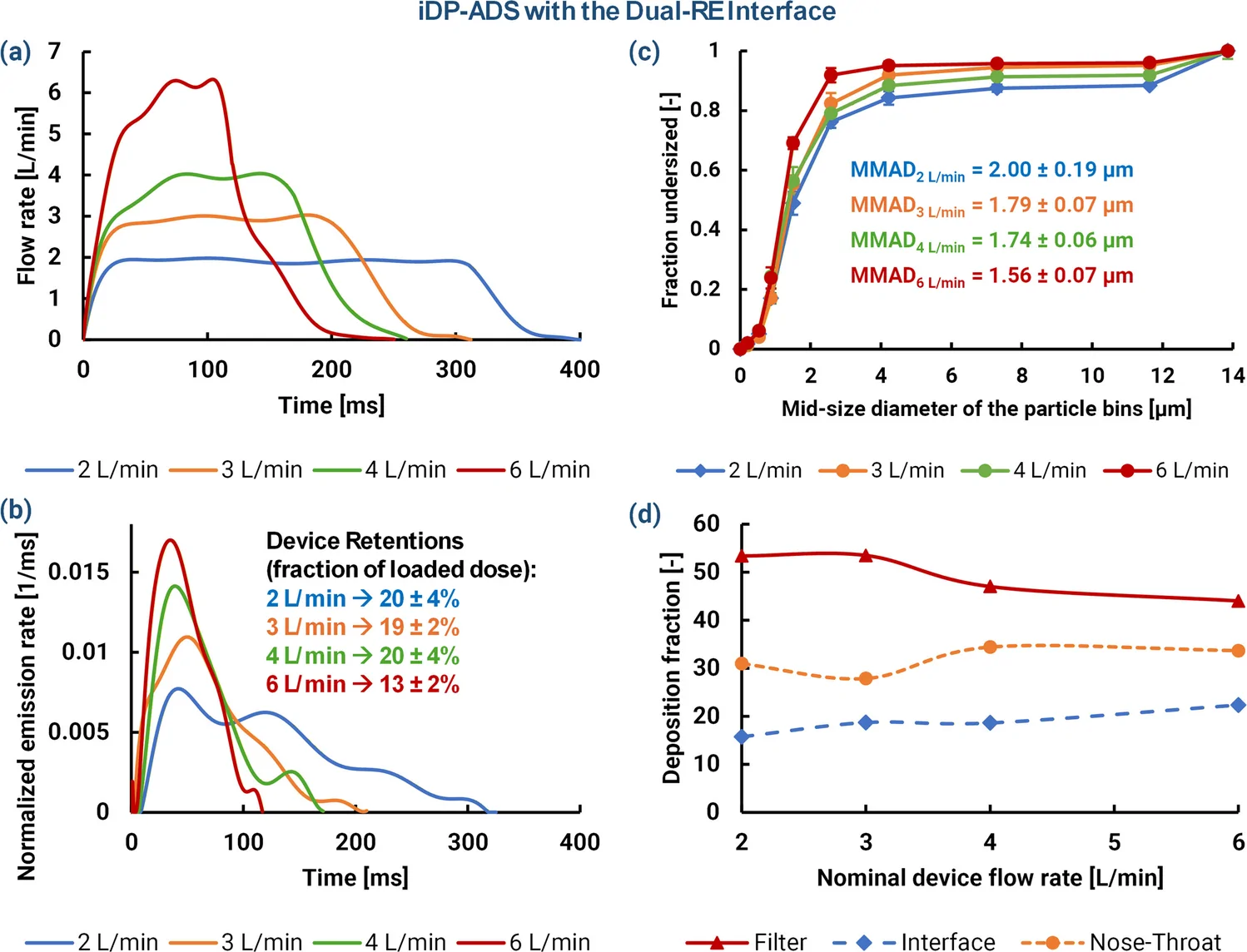
Impact of device actuation flow rate on dual-RE interface system performance. (**a**) Measured device actuation flow rate profiles investigated in this section. (**b**) Corresponding aerosol emission profiles generated by the aerosolization engine of the iDP-ADS. (**c**) Emitted particle size distributions at varying flow rates measured by the NGI. (**d**) Regional deposition fractions demonstrating the effect of flow rate on lung delivery performance.

This study evaluated four nominal actuation flow rates ranging from 2 to 6 L/min in the dual-RE system. To maintain a constant total actuation volume of approximately 10 mL, actuation durations varied inversely with flow rate, ranging from 400 ms at 2 L/min to approximately 133 ms at 6 L/min. Comparison of the recorded flow rate profiles (Fig. 9a) reveals that the desired square waveform—characterized by rapid ramp-up/down phases and a sustained peak flow—is achieved more effectively by the EM timer at lower flow rates. Consistent with the front-loading design objective discussed in the previous section, the iDP-ADS aerosolization engine demonstrated a consistent capability to emit the majority of the particle mass within the first half of the actuation period across all tested rates (see Fig. 9b). However, increasing the flow rate led to a sharper and more intense initial emission peak, reflecting enhanced early-stage powder fluidization and dispersion under higher shear and turbulence levels. Furthermore, increasing the flow rate to 6L/min resulted in improved device emptying compared to lower flow rates. Device retention fractions were comparable at 2, 3, and 4 L/min (20 ± 4%, 19 ± 2%, and 20 ± 4%, respectively) but dropped to 13 ± 2% at 6 L/min, indicating a significant improvement in dose emission at the highest tested flow rate.

The aerodynamic impact of flow rate on particle size distributions was evident, with the PSD shifting toward smaller diameters as flow rate increased (Fig. 9c). Specifically, the MMAD demonstrated a negative correlation with flow rate, decreasing from 2.0 μm at 2 L/min to 1.56 μm at 6 L/min. Concurrently, the Fine Particle Fraction (FPF) increased; specifically, the $FP{F}_{\le 5\upmu m}$ rose from 85.1% to 95.3%, and the sub-micron fraction ($FP{F}_{\le 1\upmu m}$) increased from 23.4% to 33.0%. It is noteworthy that while tripling the flow rate produced measurable shifts in sizing, the variation was relatively moderate (approximately 25% for MMAD and 10% for FPF). This stability, combined with low standard deviations (n = 3), highlights that the aerosolization engine maintains robust and repeatable dispersion performance across a clinically relevant flow range. Furthermore, comparison of cumulative distributions reveals that the span of the PSD generally narrows at higher flow rates, decreasing from 6.5 at 2 L/min to 1.6 at 6 L/min. The intermediate flow rates of 3 and 4 L/min yielded spans of 3.5 and 4.1, respectively. The presence of a larger coarse fraction at 2 L/min suggests that lower shear forces are less effective at de-agglomerating the powder formulation, whereas the elevated turbulent intensity at higher velocities enhances de-agglomeration efficiency and limits the persistence of larger particle clusters.

These flow-dependent changes in emission dynamics and PSD exert a complex, compounded effect on regional deposition in D2I transnasal AST. CFD predictions (see Fig. 9 d) indicate that depositional losses within the PI are generally proportional to the flow rate, rising consistently from 15.7% at 2 L/min to 22.3% at 6 L/min. This trend aligns with the established dominance of turbulent dispersion within the interface, as higher flow rates intensify turbulent kinetic energy and particle–eddy interactions.

In contrast, depositions in the NT region exhibited non-monotonic behavior. Total NT deposition decreased from 30.9% at 2 L/min to a minimum of 27.8% at 3 L/min, before rising to 34.4% at 4 L/min and stabilizing at 33.7% at 6 L/min. This behavior can be explained by the competing effects of particle inertia and impaction velocity. The phenomenon of impaction loss is fundamentally governed by the Stokes number (Stk), which dictates that deposition via inertial impaction is proportional to the carrier gas velocity (or flow rate, Q) and the square of the particle diameter (${d}^{2}$). Because the CFD simulations utilized the mean measured PSDs for each flow rate, the model deterministically reflects the average shifts in particle size without accounting for experimental standard deviations. Based on these mean inputs, the transition from 2 to 3 L/min led to reduced particle size (MMAD 2.00 → 1.79 μm), modestly lowering particle inertia to partially offset the increase in impaction velocity. Evaluated via the impaction parameter (${d}^{2}Q$), this size reduction does not completely offset the 1.5-fold increase in flow rate, yielding a modest increase (~ 20%) in impaction potential. However, this slight increase in NT impaction propensity is outweighed by a concurrent rise in upstream interface loss. This upstream deposition effectively acted as a filter, reducing the mass fraction of particles available to enter the downstream geometry and driving the net reduction in NT deposition. Conversely, at 4 L/min, the increased gas velocity seemingly outweighed the benefit of reduced particle size, particularly because the incremental reduction in size (MMAD 1.79 → 1.74 μm) was comparatively small. This drove NT deposition up to its maximum value of 34.4%, primarily due to increased impaction losses in the anterior nasal region (22.8% to 28.5%). At 6 L/min, these counteracting effects appeared to reach an equilibrium, keeping NT deposition relatively constant.

The net effect of these competing mechanisms is reflected in lung transmission, measured as DF in the tracheal filter. Lung transmission remained virtually stable between 2 and 3 L/min (53.4% and 53.5%, respectively) before declining significantly to 47.0% at 4 L/min and 44.0% at 6 L/min. This initial plateau suggests an operational optimum at 3 L/min. While total system efficiency was nearly identical between 2 and 3 L/min, the 3 L/min condition not only produced finer aerosol sizes—which are more likely to reach the lower airways—but also shifted a portion of the unavoidable loss from the nasal cavity to the removable PI. From a clinical perspective, this shift is advantageous. As discussed regarding the optimization of interface length, minimizing off-target deposition in the nasal airways is critical for surfactant administration to prevent airway obstructions.

Overall, these findings reveal that actuation flow rate is a crucial parameter with complex, often opposing effects on delivery variables. While higher flow rates appear to improve aerosolization performance (lower MMAD, higher FPF, narrower PSD) alongside shortening the administration duration, these benefits can be largely negated by increased depositional losses in the interface as well as in the upper airways, ultimately reducing lung transmission. While the identified optimum flow rate of 3 L/min is specific to the D2I transnasal AST with the iDP-ADS and the SLS-EEG formulation evaluated here, the broader implication is that maximizing drug delivery efficiency requires system-level optimization rather than isolated aerodynamic improvement. Effective device design must therefore consider the balance between aerodynamic dispersion efficiency and transport losses governed by distinct physical mechanisms across different regions of the system. Furthermore, the feasible optimization window must remain clinically constrained, accounting for factors such as acceptable breath delivery durations and potential consequences of regional distribution of depositional losses.

### Limitations

While the CFD predictions demonstrated excellent agreement with the regional *in vitro* data, several modeling simplifications were employed. An airtight seal was assumed between the PI and the NT model, a condition supported by the high experimental mass recovery (≥ 95%). The CFD simulations utilized one-way momentum coupling; two-way momentum coupling between the dispersed and continuous phases was not considered because the aerosol loading was dilute (volume concentration < 0.05%), rendering the particle effects on the continuous flow field negligible. In addition, several discrete-phase phenomena were omitted, including inter-particle interactions (e.g., collisions, agglomeration, and break-up), particle re-entrainment, and large particle rebound from walls. Hygroscopic growth was also excluded because the combination of a very short particle residence time (< 15 ms) and low humidity of the actuation gas (~ 20% or less RH) prevents significant growth of the EEG formulation within the nasal airways, with corresponding minimal change in depositional loss. Additional transport mechanisms, including lift forces, particle shape effects, and Brownian motion (which is negligible for particles > 100 nm [67]), were not included based on preliminary assessments and prior studies demonstrating a minimal influence on deposition relative to turbulent dispersion. Electrostatic effects were likewise not considered because sufficient charge characterization data were unavailable. Despite these simplifications, the strong agreement between CFD predictions and experimental measurements suggests that the adopted assumptions were appropriate for the objectives of the present study.

Owing to the scope of this work, which focused on aerosol transport through the PI and upper airways, the model did not include post-deposition processes such as dissolution, spreading and absorption of the formulation. Future studies incorporating surface liquid tracking [e.g., 68, 69, 70] will be valuable for determining the local concentration and liquid-phase spreading of the surfactant. Moreover, the reported lung transmission values represent the fraction of aerosol exiting the modeled upper airways. These values should not be interpreted as the dose delivered to the alveolar region. The aerosol transmitted beyond the modeled geometry will undergo additional transport and deposition within the conducting and peripheral airways, while a portion may subsequently be exhaled, potentially contributing to additional airway deposition during the expiratory phase. Therefore, the present study quantifies delivery beyond the upper airways rather than the final deposited dose within the deep lung. Future whole-lung models incorporating the lower airway architecture and simulations encompassing the entire breathing cycle will be necessary for determining the true dose retained in the alveolar region. A further limitation is that the analysis was conducted using a single standardized 1500 g reference preterm NT geometry to extract the lessons and systematically isolate device-level variables. However, the underlying computational methodology is not specific to this geometry. Following its development and validation against both benchmark and complex aerosol delivery cases [47], the framework has been successfully applied across a wide range of infant, pediatric, adult, and animal airway models [41, 69, 71], with study-specific validation against *in vitro* data. Thus, the broad conclusions derived here are based on universal fluid-particle transport mechanics. Finally, while the baseline model was rigorously validated experimentally, the subsequent design modifications were evaluated purely *in silico*. Although we have high confidence in the predictive capabilities of the CFD framework, broad conclusions drawn exclusively from computational predictions carry inherent risk. Physical prototyping and *in vitro* corroboration of these optimized designs, alongside testing across a wider cohort of subject-specific neonatal airways, remain important next steps to definitively confirm the performance improvements of the iDP-ADS identified in this study.

## Conclusions

This study employed CFD to systematically examine the modeling and design space of direct-to-infant (D2I) transnasal aerosol surfactant therapy (AST) with the infant dry powder aerosol delivery system (iDP-ADS). After validation against experimental data, including assessment of common simplifications such as monodisperse distributions, steady-state approximations, and exclusion of turbulent dispersion, the digital twin was used to evaluate controllable design and operating parameters affecting delivery performance. Sensitivity analyses examined interface geometry, flow development, interface alignment, and aerosol emission characteristics, elucidating mechanisms behind particle transport and deposition, quantifying their differential contributions, and identifying design trade-offs.

The key lessons learned include:

1. **Modeling fidelity**: Common modeling simplifications—steady-state solvers and monodisperse particles—overpredicted lung transmission (~ 24% for the steady-state approximation and 4.3% for the monodisperse approximation). Fully transient simulations with polydisperse distributions—incorporating time-varying data where available—are required for capturing the flow and dispersion physics unique to air-jet DPIs, although simplified models remain useful for trend identifications.
2. **Interface including upstream volume**: Recirculation, often seen as a liability, can be an aerodynamic advantage; a simple cylinder upstream of the high-velocity inlet jet creates controlled recirculation that shields against particle–wall interactions in the jet expansion region, cutting interface losses by up to 50%.
3. **Trade-offs associated with geometric modifications**: Geometric modifications produced trade-offs between interface and NT deposition. Increasing the length of the jet diffusion element reduced nasal deposition by dissipating jet momentum and turbulence, but increased interface deposition due to larger surface area. A length (10 mm for this system) balances this trade-off, while longer elements can shift unavoidable deposition away from non-target anatomical regions to the removable interface, if clinically warranted. Furthermore, while internal anatomy is fixed, external prong alignment (20° inward and downward) can direct the flow to minimize impaction losses, particularly for larger particles or higher flow rates.
4. **Mechanism-specific targeting**: Turbulent dispersion dominates interface losses, while inertial impaction drives nasal losses, requiring distinct design strategies. Turbulence model selection should likewise be tailored to the dominant deposition mechanism to ensure predictive accuracy [72].
5. **Non-monotonic impacts of actuation flow rates**: Flow rate simultaneously governs aerosolization, emission timing, particle size, and deposition. Higher rates improve deagglomeration and produce finer size distributions favoring deep lung delivery, but also raise velocity, turbulence, and depositional losses. Because these competing effects scale disproportionately across different regions of the system, the net lung transmission varies non-monotonically with flow rate. A system-specific optimum at 3 L/min balances the aerosolization efficiency against transport losses.
6. **Aerosol emission timing**: Interface geometry alone cannot maximize delivery efficiency; the timing of aerosol release is equally important. Front-loaded release with the remaining actuation volume serving as “chase air” reduced dead-space particle retention, which otherwise accounted for approximately 10–14% of the emitted dose, indicating the emission profile should be synchronized with the beginning of the inhalation phase and ideally the first 25–50% of the inhalation breath.
7. **The value of CFD**: Beyond serving as a predictive surrogate for experiments, CFD provided a diagnostic framework for identifying transferable design insights. By isolating specific key physical phenomena—such as decoupling turbulent dispersion from mean flow transport (quantified as 29.1% depositional losses), visualizing the hydrodynamic shielding effect of upstream volumes, and jet dissipation behavior—CFD provided mechanistic explanations often inaccessible experimentally. These insights apply across interfaces and AST platforms, making validated CFD-models a critical, cost-effective tool for accelerating respiratory drug delivery development.

In summary, high-efficiency aerosol delivery is achieved not through a single iDP-ADS feature, but by balancing fluid dynamics, geometry, and clinical applicability. The lessons extracted here based on CFD simulations and related to jet conditioning, turbulence, alignment, actuation flow rate, emission timing, and particle transport will guide future advancement of the D2I transnasal AST.

## Supplementary Information

Below is the link to the electronic supplementary material.

Supplementary File 1 (MP4 5.67 MB)

Supplementary File 2 (MP4 7.52 MB)

## Data Availability

Supporting data are openly available on OSF: https://osf.io/bzs54/overview?view_only=97333359ef7b44409ba33599dfec1010.

## Abbreviations

AST: Aerosol Surfactant Therapy
CFD: Computational Fluid Dynamics
CPAP: Continuous Positive Airway Pressure
D2I: Direct-to-Infant
DF: Deposition Fraction
DPI: Dry Powder Inhaler
DPM: Discrete Parcel Method / Discrete Phase Model
DPPC: Dipalmitoylphosphatidylcholine
EEG: Excipient Enhanced Growth
EIM: Eddy Interaction Model
EM: Electromechanical
HPLC: High Performance Liquid Chromatography
ID: Internal Diameter
iDP-ADS: Infant Air‑Jet Dry Powder Aerosol Delivery System
LRN: Low Reynolds Number
MMAD: Mass Median Aerodynamic Diameter
nCPAP: Nasal Continuous Positive Airway Pressure
NGI: Next Generation Impactor
NT: Nose-Throat
PEEP: Positive End-Expiratory Pressure
PI: Patient Interface
PIP: Peak Inspiratory Pressure
PMC: Pressure Monitoring and Control
POPG: 1-Palmitoyl-2-oleoyl-sn-glycero-3-phosphoglycerol
PRESTO: Pressure Staggering Option
PSD: Particle Size Distribution
RDS: Respiratory Distress Syndrome
RE: Rapid Expansion
SD: Standard Deviation
SIMPLEC: Semi-Implicit Method for Pressure Linked Equations-Consistent
SLA: Stereolithography
SLS: Synthetic Lung Surfactant
SST: Shear Stress Transport
TKE: Turbulent Kinetic Energy
TPP: Target Product Profile
